# Eugenol as a potential adjuvant therapy for gingival squamous cell carcinoma

**DOI:** 10.1038/s41598-024-60754-8

**Published:** 2024-05-13

**Authors:** Hawraa Issa, Lionel Loubaki, Abdullah Al Amri, Kazem Zibara, Mikhlid H. Almutairi, Mahmoud Rouabhia, Abdelhabib Semlali

**Affiliations:** 1 GREB Research Group, Faculty of Dentistry, Laval University, Québec, Canada; 2 Héma-Québec, Medical Affairs and Innovation, Québec, Canada; 3 Biochemistry Department, College of Science, King Saud University, Riyadh, Saudi Arabia; 4 PRASE and Biology Department, Faculty of Sciences-I, Lebanese University, Beirut, Lebanon; 5 Zoology Department, College of Science, King Saud University, Riyadh, Saudi Arabia

**Keywords:** Oral cancer, Gingival and tongue carcinomas, Eugenol, Phytotherapy, Tumorigenesis, Cancer, Cell biology, Chemical biology, Molecular biology

## Abstract

Adoption of plant-derived compounds for the management of oral cancer is encouraged by the scientific community due to emerging chemoresistance and conventional treatments adverse effects. Considering that very few studies investigated eugenol clinical relevance for gingival carcinoma, we ought to explore its selectivity and performance according to aggressiveness level. For this purpose, non-oncogenic human oral epithelial cells (GMSM-K) were used together with the Tongue (SCC-9) and Gingival (Ca9-22) squamous cell carcinoma lines to assess key tumorigenesis processes. Overall, eugenol inhibited cell proliferation and colony formation while inducing cytotoxicity in cancer cells as compared to normal counterparts. The recorded effect was greater in gingival carcinoma and appears to be mediated through apoptosis induction and promotion of p21/p27/cyclin D1 modulation and subsequent Ca9-22 cell cycle arrest at the G0/G1 phase, in a p53-independent manner. At these levels, distinct genetic profiles were uncovered for both cell lines by QPCR array. Moreover, it seems that our active component limited Ca9-22 and SCC-9 cell migration respectively through MMP1/3 downregulation and stimulation of inactive MMPs complex formation. Finally, Ca9-22 behaviour appears to be mainly modulated by the P38/STAT5/NFkB pathways. In summary, we can disclose that eugenol is cancer selective and that its mediated anti-cancer mechanisms vary according to the cell line with gingival squamous cell carcinoma being more sensitive to this phytotherapy agent.

## Introduction

Oral neoplasms are ranked as the 16th most common malignancies worldwide, with almost 355,000 new cases per year^1^. Oral cancer is frequent in men after the fifth decade of life with squamous cell carcinoma accounting for more than 90% of all oral cancers^2^. The main etiological factors include among others tobacco smoking, betel quid and areca nut chewing, alcohol use, poor oral hygiene, diet plan and genetic conditions^3–5^. Despite easy self-examination through visual inspection and palpation, patients often present with advanced stage disease, as early carcinomas are often asymptomatic^1^. Diagnosis by biopsy followed by preoperative radiographic imaging is essential for staging and decision-making^6^. On this level, the NCCN guidelines offer a detailed overview for care recommendations according to disease stage and pathological findings^7^. Despite proximity, the affected oral cavity subsite is also to be considered when planning therapy due to distinct anatomical features. In general, the standard oncologic control procedure consists of primary surgical resection^8^. According to tumor size and location, invasive approaches become necessary and involve a multidisciplinary team of experts to ensure favorable outcomes through reconstruction of surgical defects together with speech, swallowing and behavioral rehabilitation. As for postoperative adjuvant therapy, it is recommended for patients with high risk of locoregional recurrence^9^. Although the traditional modality is based on the use of radiation, concurrent administration of chemotherapy agents improves locoregional control and survival in head and neck cancer patients^10^. Considering that the overall 5 years survival rate is 56%^2^ and that 10–40% of the patients may develop metachronous tumors in the first decade after treatment completion^10^, growing efforts are directed towards implementation of comprehensive therapeutic plans to improve prevention and treatment of this type of cancer.

Cisplatin, also named cis-diaminedichloroplatinum, is considered among the first-line and most effective chemotherapy drug used for the management of oral cancer through generation of DNA lesions and cell apoptosis^11,12^. However, being nonselective for cancer cells, many side effects present in the form of bone marrow suppression, hair loss and nausea^13,14^. Henceforward, the current focus is leaning towards phytochemicals due to reported effectiveness and reduced adverse effects^13,15^. In fact, the use of herbal remedies by ancient civilisations led to characterization of active principles that increased the pace of drug discovery. Over the period between 1981 and 2014, natural products served as precursor for more than half of all approved small‐molecule drugs^16^. Previous studies have demonstrated that natural compounds and their derivatives or analogs were also found to regulate main molecular pathways implicated in cancer growth and progression through antioxidant status stimulation, autophagy enhancement, carcinogen inactivation, invasiveness hindering, angiogenesis blockade, proliferation control, cell cycle arrest, apoptosis stimulation and modulation of the immune response^17,18^. Another major issue that can be handled by the use of natural products is the emergence of clinical resistance following administration of high doses chemotherapeutic agents. At this echelon, promising synergistic effects were recorded when combining phytochemicals with the cisplatin chemotherapy agent in preclinical settings^19,20^. Despite the progress observed, various phytochemicals are yet to undergo initial clinical testing^21–25^.

Eugenol (4-allyl-2-methoxyphenol) is a biologically active phenolic compound found in aromatic plants including cloves, nutmeg, basil, and cinnamon^26^. It has been traditionally used in perfumeries, essential oils, and flavorings^27^. It also has some applications in medicine as an antiseptic, anesthetic, analgesic, antibacterial, anti-viral, and a cavity filling cement^26,27^. Its safety as well as its pharmacokinetic proprieties have been addressed prior to its release in the market^28–30^. As compared to other phytochemicals, eugenol stands out as a candidate for cancer treatment due to its availability, effectiveness in multiple in vitro and in vivo models, multi-targeted therapeutic outcomes alongside with its synergistic potential^31^. In greater detail, drug effectiveness has been extensively studied and covers melanoma, skin cancer, osteosarcoma and leukemia among other types of tumors^32^. On the other hand, this nutraceutical agent has been reported to possess antioxidant, anti-inflammatory, anti-genotoxic, anti-mutagenic, anti-angiogenic, anti-metastatic, anti-proliferative and pro-apoptotic activities^33,34^. Moreover, it was shown to attenuate cisplatin mediated toxicity and to sensitize of cisplatin-resistant cells by triggering apoptosis. More in depth, eugenol-cisplatin combinations showed great therapeutic value in ovarian and breast tumor bearing mice and this is in terms of growth, inflammation, epithelial-to-mesenchymal transition, disease-free survival and cancer stem cells self-renewal^35,36^.

In relation to oral cancers, eugenol was showed to supress tongue carcinoma malignant processes in vitro. In particular, eugenol hindered cell proliferation, colony formation, invasion and migration while stimulating SCC-9 tongue carcinoma apoptosis by targeting the macrophage migration inhibitory factor expression^37^. Surducan et al., also revealed increased expression of pro-apoptotic genes and apoptotic-like indicators in SCC-4 cell line treated with eugenol^38^. Apoptosis induction and S-phase arrest were also reported at the level of the SCC-25 cell line^39^. One paper suggested that this product rather alters the metabolic profile and favors non-apoptotic cell death^40^. To our knowledge, no report leaded in-depth investigation on the specific management of gingival carcinoma by eugenol. Thus, we ought to explore its clinical relevance in vitro on Ca9-22 gingival carcinoma cell line. Being considered as a more aggressive entity, SCC-9 tongue carcinoma were also utilized to confirm treatment efficacy according to aggressiveness level. Eugenol selectivity was tested in the presence of GMSM-K non-oncogenic human oral epithelial cell lines. Overall, our first target was to confirm drug effect on cancer cell proliferation and colony formation while excluding any potential cytotoxic effect. Afterwards, it was important to link the observed outcomes to either a blockade of the cell cycle or an induction of cell death by apoptosis. To better understand eugenol mechanisms of actions, our study also addressed its potential impact on migration as well as on key oncogenesis signaling pathways.

## Materials and methods

### Cell culture

Ca9-22 Human gingival carcinoma cell line (RIKEN BioResource Research Center, CVCL_1102), isolated from a 43-year-old Japanese male patient with gingival squamous cell carcinoma, was cultured in RPMI 1640 medium (Gibco, 31800089) supplemented with 5% Fetal Bovine Serum (FBS) (Gibco, 12483-020), 0.2% Penicillin/Streptomycin (P/S) (Sigma-Aldrich, P4333) and 0.2% Fungizone (F) (Sigma-Aldrich, A2942). On the other hand, the SCC-9 Human tongue squamous carcinoma cell line (ATCC, CRL1629) took origin from a 25-year-old male patient with primary tongue carcinoma. Non-oncogenic human oral epithelial cells, derived from a 30-week gestational stillborn male fetus, were designated as GMSM-K^41^ (cell line provided by Dr. Daniel Grenier, Laval University, Québec city, Canada). Both cell lines were maintained using the DMEM/F12 medium (Gibco, 11320033) supplemented with 10% FBS together with 0.2% P/S and 0.2% F. All cells were exposed to different concentrations of eugenol (MedChemExpress, HY-N0337) for 24 h.

### MTT proliferation and viability assay

The cells were seeded in 24 well plates and incubated with the 3-(4,5-dimethylthiazol-2-yl)-2,5-diphenyltetrazolium bromide MTT agent (Sigma-Aldrich, M-2128) diluted at 0.5 mg/ml for 3 h. The plates were then covered and kept in the incubator at 37 °C to allow reduction of the yellow tetrazolium salt to purple formazan crystals by metabolically active cells. The insoluble crystals were dissolved by using the isopropanol 0.4% HCl and the optic density was measured at 550 nm using the Bio-Rad xMark™ Microplate Absorbance Spectrophotometer. IC50 is defined as the half maximal inhibitory concentration required to inhibit a biological process by half. Three technical replicates and four biological replicates were carried out.

### LDH cytotoxicity assay

The Lactate dehydrogenase LDH kit (Roche, 11644793001) was used according to the manufacturer’s instructions to assess cytotoxicity levels. Dye and catalyst solutions were added to culture supernatants for 30 min. The formation of the red formazan product being directly proportional to the enzyme released ensured quantification of the extracellular LDH released upon damage to plasma membrane. Optic density was measured at 490 nm by the means of the Bio-Rad xMark™ Microplate Absorbance Spectrophotometer. Three technical replicates and four biological replicates were carried out.

### Colony formation assay

The cells were grown for 14 days to allow colony formation in the presence or absence of different concentrations of eugenol then they were rinsed twice with PBS before being fixed with cold methanol for 10 min. 1% Crystal violet (Sigma-Aldrich, 548-62-9) was added for another 10 min and the excess of the product was washed away using water. Colonies were photographed to show differences at this level. Four biological replicates were carried out.

### Annexin V/propidium iodide apoptosis test

Following treatment with eugenol, dead cells populations were analyzed using the APC Annexin V apoptosis detection kit with PI (Biolegend, 640932). After 24 h of eugenol treatment, the apoptosis and necrosis markers—annexin V (AnxV) and propidium iodide (PI)—were incubated with the cells for 20 min at room temperature. Analysis with the BD FACSCanto II flow cytometry system permitted events classification as viable (AnxV−/PI−), early apoptotic (AnxV+/PI−), late apoptotic (AnxV+/PI+) and necrotic cells (AnxV−/PI+). Three biological replicates were carried out.

### Western blot

Protein extraction was performed using a RIPA Lysis buffer supplemented with protease and phosphatase inhibitors cocktails (Sigma-Aldrich, P0044) while protein concentration was determined using the Bradford assay (Bio-Rad, 5000006). Briefly, western blot samples were exposed to denaturation then migrated through 8, 12 and 15% polyacrylamide gels. Proteins were transferred to nitrocellulose membranes and 5% milk solution was used for blocking purposes. The primary antibodies were added overnight after being diluted in 1% milk solution: p53 (Santa cruz, sc263, 1:200), p21 (Santa cruz, sc6246, 1:100), p27 (Santa cruz, sc71813, 1:100), cyclin D1 (Santa cruz, sc8396, 1:200), Noxa (Santa cruz, sc515840, 1:100), PARP1 (Santa cruz, sc8007, 1:200), GAPDH (Santa cruz, sc47724, 1:1000). Membranes were then incubated with the goat anti-mouse HRP conjugate secondary antibody (Bio-Rad, 1706515, 1:2000) prepared in 1% milk solution and this for 1 h before being exposed to the Clarity western ECL substrate (Bio-Rad, 1705061). Band detection was enabled using the Bio-Rad VersaDoc Imaging system and quantification was done by the means of the ImageJ software. Three to four biological replicates were carried out.

### Polymerase chain reaction

Total RNA was extracted using the Rneasy mini kit (Qiagen, 74104) according to manufacturer’s recommendations while concentration and purity were assessed with the ThermoFisher nanodrop 8000 spectrophotometer. 2 µg total RNA were reverse transcribed into cDNA copies using the iScript™ Reverse transcription Supermix for RT-qPCR (Bio-Rad, 1708841). For Real time PCR, the following primers sequences were adopted (pro-MMP1 (F) 5′-GATCATCGGGACAACTCTCCT-3′ and ® 5′-TCCGGGTAGAAGGGATTTGTG-3′, pro-MMP3 (F) 5′-CACTGTCCACCCTCAGAGC-3′ a®(R) 5′-GCCACTTGTCGGCGATAAGG-3′, GAPDH (F) 5′-ATGCAACGGATTTGGTCGTAT-3®nd (R) 5′-TCTCGCTCCTGGAAGATGGTG-3′) along with the IQ™ SYBR Green supermix (Bio-Rad, 64204590). Transcript levels were analysed with the Bio-Rad CFX Manager 3.1 software and normalized to GAPDH. Relative gene expression data analysis was performed using the 2-∆∆CT method. Three technical replicates and four to six biological replicates were carried out.

### QPCR arrays

RT^2^ Profiler PCR arrays corresponding to human cell cycle (Qiagen, PAHS-020ZD-6) and apoptosis (Qiagen, PAHS-012ZD-6) served to unravel key determinants implicated in cancer progression. The acquired data was analyzed using the 2-ΔΔCT method to determine relative gene expression and the fold changes between non-exposed and eugenol-treated cells. A gene was considered upregulated or downregulated when the fold change exceeded twice the initial value. CT values were extracted and compiled into a table, which was subsequently uploaded to the web portal for data analysis, accessible at http://www.qiagen.com/geneglobe. The values obtained were normalized based on an automated selection from a comprehensive set of reference genes. This indicative study corresponds to only one experiment.

### Cell cycle distribution

Cells were treated with different concentrations of eugenol for 24 h then exposed to the action of trypsin. Harvested cells were washed, fixed in cold 70% ethanol for 1 h then exposed to the action of ribonucleases (Roche, 93137524) to ensure only the DNA is stained by propidium Iodide (Biolegend, 640932). The later was incubated with the cells for 20 min at room temperature. Analysis using the BD FACSCanto II flow cytometry system allowed classification according to the cell cycle phase. Three biological replicates were carried out.

### Scratch assay

A simple scratch was created in cell monolayer and the capacity of the cells to migrate in the presence of different eugenol concentrations was studied. Images were taken with the Nikon ECLIPSE TS100 optical microscope at two time points: the beginning of the experiment then after 24 h. The microscope magnification was set at × 10. At the end point, cells were stained with crystal violet as previously described to obtain better quality photos under the microscope. Scratch diameter was measured using the Lumenera infinity analyze 6.5.5 software to quantify the scratch healing process. Three to four biological replicates were carried out.

### Gelatin zymography

Polyacrylamide gels containing 1% gelatin (J.T.Baker, 424865) allowed to screen for MMP-2 and MMP-9 gelatinase enzymes in cell supernatants. Samples were prepared in a standard non reducing buffer and subjected to electrophoresis. SDS was removed from the gels by the action of the 2.5% triton X-100 washing solution. The later was replaced by the developing buffer so that gelatin digestion can occur overnight at 37 °C. Gels were then stained following 30 min exposure to Coomassie Brilliant Blue R-250 (Bio-Rad, 161-0436) and bands detection was warranted after incubation with the de-staining buffer consisting of 25% methanol 10% acetic acid. Three to four biological replicates were carried out.

### Flow cytometry analysis of signaling pathways

As previously described by our team^25^, cells were washed then fixed in 1.5% paraformaldehyde for 20 min at room temperature. Cells were re-rinsed and permeabilized using a 90% methanol/PBS solution. This step was realized on ice for a total period of 20 min. An additional wash cycle was performed, and cells were labeled for 30 min using the following fluorescence conjugated primary antibodies: pSTAT1 (BD Biosciences, AB_1645373), pSTAT3 (BD Biosciences, AB_647232), pSTAT5 (BD Biosciences, AB_399858), pERK1/2 (BD Biosciences, AB_399857), phosphorylated p38 (BD Biosciences, AB_399856) and Phospho NF-κB p65 (Thermofisher, AB_2572751). Cells were washed for the last time and flow cytometry analysis using the BD Accuri C6 Plus flow cytometer allowed estimation of ERK1/2, NF-κB, STAT1, STAT3, STAT5 and P38 phosphorylation levels. The histogram subtraction technique was applied based on Overton subtraction and thus the overlay of the histograms of interest. The analysis was then realized by the FCS express De Novo software^42^.Three biological replicates were carried out.

### Statistical analysis

The prism software 9.0.0 version was used. Unpaired t Test, one-way and two-way Anova ensured results analysis. *p < 0.05, **p < 0.01, ***p < 0.001 and ****p < 0.0001 were considered as statistically significant.

## Results

### Eugenol induces cytotoxicity and hinders cell proliferation as well as colony formation

**Figure 1:**
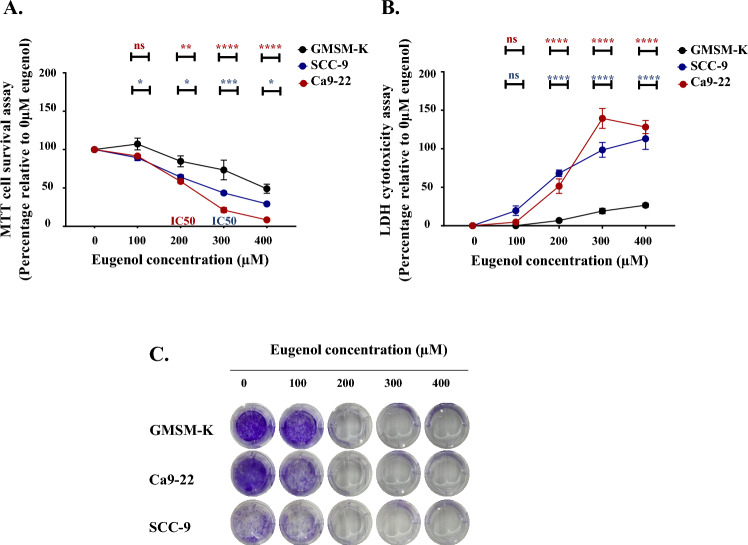
Effect of eugenol on cell viability, proliferation and colony formation. (**A**) MTT cell proliferation and viability test (n = 4) and (**B**) LDH cytotoxicity assay (n = 4) carried out after treatment of Ca9-22 and SCC-9 human carcinoma cell lines with different concentrations of eugenol (0, 100, 200, 300 and 400 µM) for 24 h. Comparisons are presented relative to corresponding GMSM-K controls. (**C**) Crystal violet staining (n = 4), performed 14 days post eugenol administration, to allow estimation of colony formation capacity. All presented data are expressed as mean values ± SEM of four independent experiments. *p < 0.05, **p < 0.01, ***p < 0.001 and ****p < 0.0001 were considered as statistically significant.

Eugenol treatment counteracts Ca9-22 and SCC-9 proliferation in a dose dependent manner with gingival cell line being more sensitive to treatment. This is clearly showing following supplementation of high eugenol concentrations. IC50, also known as the half maximal inhibitory concentration, is recorded around 200 µM for Ca9-22 and 300 µM for SCC-9. Interestingly, our phytotherapy agent was unfolded as cancer selective (Fig. 1A). This is also showing at the level of the LDH assay where little cytotoxicity was linked to the GMSM-K normal cells as compared to the Ca9-22 and SCC-9 models (Fig. 1B). Moreover, crystal violet staining demonstrated complete abolition of colony formation at concentrations as low as 200 µM. In this respect, a repressive effect is detected at 100 µM for Ca9-22 (Fig. 1C).

### Eugenol exhibits its effects on gingival carcinoma through cyclin D1 modulation and subsequent cell cycle arrest at the G0/G1 phase

**Figure 2:**
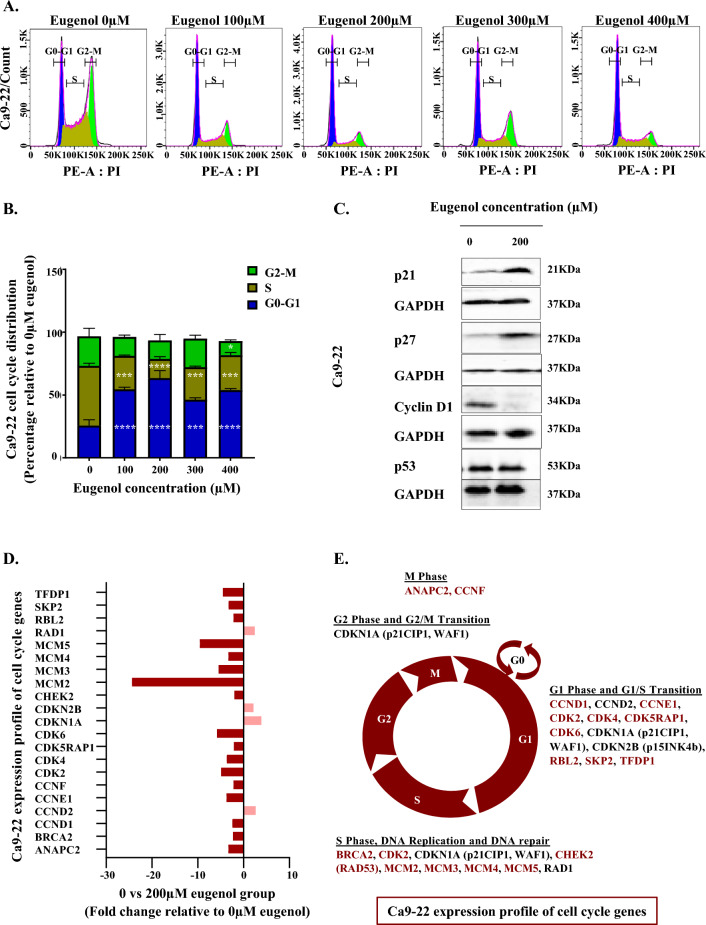
Effect of eugenol on cell cycle progression. (**A**) and (**B**) Flow cytometry results showing Ca9-22 cell cycle phases distribution following treatment with different concentrations of eugenol for 24 h (n = 3). For each phase of the cell cycle, comparison was done relative to the 0 µM eugenol control condition. (**C**) This was complemented by the examination of certain cell cycle markers including p53 (n = 3), p21 (n = 4), p27 (n = 4) and cyclin D1 (n = 3) using western blot. Presented data are expressed as mean values ± SEM of three to four independent experiments. *p < 0.05, **p < 0.01, ***p < 0.001 and ****p < 0.0001 were considered as statistically significant. (**D**) Graphical representation of QPCR array data showing the effect of an intermediate concentration of eugenol, 200 µM, on Ca9-22 expression profile of cell cycle genes. The treatment time was limited to 24 h. A total number of 84 cell cycle genes were investigated. All positively and negatively modulated genes displaying at least two-fold difference relative to untreated control were presented (n = 1). (**E**) Expression profile of cell cycle genes as per the corresponding cell cycle phase. All markers highlighted in red were downregulated. This indicative study corresponds to only one experiment (n = 1).

Treatment with increasing concentrations of eugenol for 24 h favored Ca9-22 cell cycle arrest at the G0–G1 phase. In further detail, we can state that relative to the control condition, it is evident that the percentage of the cells blocked in the G0–G1 phase scored 28,9% increase when administering our phytotherapy agent at concentrations as little as 100 µM (Fig. 2A and B). The half maximal inhibitory concentration (IC50) was then used to unravel drug effect on a certain number of cell cycle genes. Western blot results disclosed that upon administration of 200 µM eugenol, no significant difference was detected at the level of the p53 tumor suppressor expression. However, the cyclin dependent kinases inhibitors (CDKIs), namely p21 and p27, were increased while cyclin D1 levels were significantly reduced (Fig. 2C, Supplementary Figs. 1 and 2). Among a total number of 84 markers, Ca9-22 profile analysis demonstrated modulation of 21 cell cycle genes upon supplementation of eugenol 200 µM for 24 h. Changes below two-folds were not taken into consideration for this indicative study. Overall, four markers namely cyclin D2 (2.63 fold), p21 (3.87 fold), p15 (2.13 fold) and RAD1 (2.43 fold) were upregulated as compared to the untreated control while the rest were negatively modulated by our phytotherapy agent. This includes the following factors: TFDP1 (− 4.52-fold), SKP2 (− 3.27 fold), RBL2 (− 2.20 fold), MCM2 (− 24.37 fold), MCM3 (− 56.45 fold), MCM4 (− 3.31 fold), MCM5 (− 9.56 fold), CHEK2 (− 2.01 fold), CDK2 (− 4.92 fold), CDK4 (− 3.67 fold), CDK5RAP1 (− 2.12 fold), CDK6 (− 5.80 fold), cyclin F (− 2.20 fold), cyclin E1 (− 3.72 fold), cyclin D1 (− 2.44 fold), BRCA2 (− 2.32 fold), ANAPC2 (− 3.31 fold) (Fig. 2D and Table 1). The modified factors corresponded to key G1 phase markers, S phase components, DNA repair agents and controllers of G1/S transition (Fig. 2E). On the other hand, it is worth noting that for the same outcome, which is eliminating half of the exposed oral cancer cells, eugenol follows a different path in tongue carcinoma cell line. More specifically, distinct profiles were uncovered for both cell lines by QPCR array and eugenol induced a blockade, most likely, post-G1 for SCC-9 (Supplementary Table 1).

**Table 1:** Effect of eugenol on Ca9-22 expression profile of cell cycle genes.

Ca9-22 expression profile of cell cycle genes				
Unigene	Refseq	Symbol	Description	Fold change
Hs. 533262	NM_013366	ANAPC2	Anaphase promoting complex subunit 2	− 3.31
Hs. 34012	NM_000059	BRCA2	Breast cancer 2, early onset	− 2.32
Hs. 523852	NM_053056	CCND1	Cyclin D1	− 2.44
Hs. 376071	NM_001759	CCND2	Cyclin D2	2.63
Hs. 244723	NM_001238	CCNE1	Cyclin E1	− 3.72
Hs. 1973	NM_001761	CCNF	Cyclin F	− 2.20
Hs. 19192	NM_001798	CDK2	Cyclin-dependent kinase 2	− 4.92
Hs. 95577	NM_000075	CDK4	Cyclin-dependent kinase 4	− 3.67
Hs. 435952	NM_016408	CDK5RAP1	CDK5 regulatory subunit associated protein 1	− 2.12
Hs. 119882	NM_001259	CDK6	Cyclin-dependent kinase 6	− 5.80
Hs. 370771	NM_000389	CDKN1A	Cyclin-dependent kinase inhibitor 1A (p21, Cip1)	3.87
Hs. 72901	NM_004936	CDKN2B	Cyclin-dependent kinase inhibitor 2B (p15, inhibits CD4)	2.13
Hs. 291363	NM_007194	CHEK2	CHK2 checkpoint homolog (S. pombe)	− 2.01
Hs. 477481	NM_004526	MCM2	Minichromosome maintenance complex component 2	− 24.37
Hs. 179565	NM_002388	MCM3	Minichromosome maintenance complex component 3	− 5.45
Hs. 460184	NM_005914	MCM4	Minichromosome maintenance complex component 4	− 3.31
Hs. 517582	NM_006739	MCM5	Minichromosome maintenance complex component 5	− 9.56
Hs. 38114	NM_002853	RAD1	RAD1 homolog (S. pombe)	2.43
Hs. 513609	NM_005611	RBL2	Retinoblastoma-like 2 (p130)	− 2.20
Hs. 23348	NM_005983	SKP2	S-phase kinase-associated protein 2 (p45)	− 3.27
Hs. 79353	NM_007111	TFDP1	Transcription factor Dp-1	− 4.52

Table presenting Unigene and Refseq database identifiers all along with genes symbols and description for all cell cycle markers showing discrepancies in response to administration of 200 µM eugenol for 24 h. A total number of 84 cell cycle genes were investigated by QPCR array and only the factors exhibiting over two-fold variation were taken into consideration. This indicative study corresponds to only one experiment (n = 1).

### Eugenol significantly induce Ca9-22 cells apoptosis

**Figure 3:**
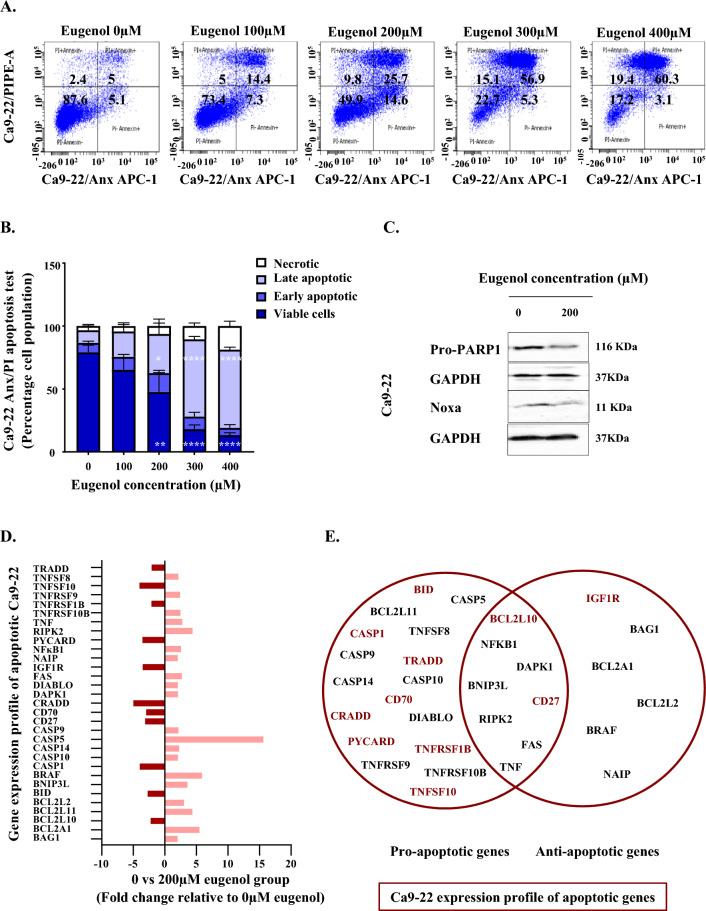
Effect of eugenol on cell apoptosis. (**A**) and (**B**) Flow cytometry data categorizing Ca9-22 cells into viable, early apoptotic, late apoptotic and necrotic based on Anx V and PI detection analysis. The cells were treated with different concentrations of eugenol for 24 h (n = 3). (**C**) Noxa (n = 4) and Pro-PARP1 (n = 3) protein expression due to the effect of 200 µM eugenol. The same time frame was adopted. Presented data are expressed as mean values ± SEM of three to four independent experiments. *p < 0.05, **p < 0.01, ***p < 0.001 and ****p < 0.0001 were considered as statistically significant. (**D**) QPCR array data corresponding to the gene expression profile of apoptotic Ca9-22. A total of 84 markers were analyzed following incubation with 200 µM eugenol for 24 h. Only the genes displaying over two-fold variation were taken into consideration (n = 1). (**E**) Recapitulation of pro- and anti-apoptotic markers modulated by eugenol. All markers highlighted in red were downregulated. This indicative study corresponds to only one experiment (n = 1).

Following supplementation of eugenol, the percentage of dead cells populations mainly the Anx V+/PI+ late apoptotic cells was significantly increased in a dose dependent manner (Fig. 3A and B). More specifically, the percentage of apoptotic cells changed from 16.8 to 86.7% upon treatment with 400 µM of eugenol. Due to the action of our polyphenol, the protein expression levels of Noxa and pro-PARP1 were significantly decreased (Fig. 3C and Supplementary Fig. 3). Based on QPCR array data, it was revealed that a total of 31 apoptotic genes are modulated by the supplementation of eugenol. More specifically, 20 factors were shown to be upregulated. This includes BAG1 (2.01 fold), BCL2A1 (5.48 fold), BCL2L2 (3.04 fold), BCL2L11 (4.36 fold), BNIP3L (3.57 fold), BRAF (5.87 fold), CASP5 (15.61 fold), CASP9 (2.15 fold), CASP10 (2.06 fold), CASP14 (2.26 fold), DAPK1 (2.06 fold), DIABLO (2.05 fold), FAS (2.68 fold), NAIP (2.03 fold), NF-ĸB1 (2.56 fold), RIPK2 (4.39 fold), TNF (2.76 fold), TNFRSF9 (2.42 fold), TNFRSF10B (2.47 fold) and TNFSF8 (2.14 fold). Among the 11 downregulated markers figures BCL2L10 (− 2.24 fold), BID (− 2.72 fold), CASP1 (− 3.96 fold), CD27 (− 3.11 fold), CD70 (− 2.96 fold), CRADD (− 4.98 fold), IGF1R (− 3.50 fold), PYCARD (− 3.54 fold), TNFRSF1B (− 2.11 fold), TNFSF10 (− 4.02 fold) and TRADD (− 2.09 fold) (Fig. 3D, E and Table 2). Importantly, discrepancies between oral carcinoma cell lines were once more detected and a different genetic profile was revealed for the SCC-9 cell line (Supplementary Table 2).

**Table 2:** Effect of eugenol on gene expression profile of apoptotic Ca9-22.

Expression profile of apoptotic Ca9-22				
Unigene	Refseq	Symbol	Description	Fold change
Hs. 377484	NM_004323	BAG1	BCL2-associated athanogene	2.01
Hs. 227817	NM_004049	BCL2A1	BCL2-related protein A1	5.48
Hs. 283672	NM_020396	BCL2L10	BCL2-like 10 (apoptosis facilitator)	− 2.24
Hs. 469658	NM_006538	BCL2L11	BCL2-like 11 (apoptosis facilitator)	4.36
Hs. 410026	NM_004050	BCL2L2	BCL2-like 2	3.04
Hs. 517145	NM_001196	BID	BH3 interacting domain death agonist	− 2.72
Hs. 131226	NM_004331	BNIP3L	BCL2/adenovirus E1B 19KDa interacting protein 3-like	3.57
Hs. 550061	NM_004333	BRAF	V-raf murine sarcoma viral oncogene homolog B1	5.87
Hs. 2490	NM_033292	CASP1	Caspase 1, apoptosis-related cysteine peptidase (interleukin 1, beta, converatse)	− 3.96
Hs. 5353	NM_001230	CASP10	Caspase 10, apoptosis-related cysteine peptidase	2.06
Hs. 466057	NM_012114	CASP 14	Caspase 14, apoptosis-related cysteine peptidase	2.26
Hs. 213327	NM_004347	CASP 5	Caspase 5, apoptosis related cysteine peptidase	15.61
Hs. 329502	NM_001229	CASP9	Caspase 9, apoptosis related cysteine peptidase	2.15
Hs. 355307	NM_001242	CD27	CD27 molecule	− 3.11
Hs. 501497	NM_001252	CD70	CD70 molecule	− 2.96
Hs. 38533	NM_003805	CRADD	CASP2 and RIPK1 domain containing adaptor with death domain	− 4.98
Hs. 380277	NM_004938	DAPK1	Death-associated protein kinase 1	2.06
Hs. 169611	NM_019887	DIABLO	Diablo, IAP-binding mitochondrial protein	2.05
Hs. 667309	NM_000043	FAS	Fas (TNF receptor superfamily, member 6)	2.68
Hs. 643120	NM_000875	IGF1R	Insulin-like growth factor 1 receptor	− 3.50
Hs. 646951	NM_004536	NAIP	NLR family, apoptosis inhibitory protein	2.03
Hs. 618430	NM_003998	NFKB1	Nuclear factor of Kappa light polypeptide gene enhancer in B-cells 1	2.56
Hs. 499094	NM_013258	PYCARD	PYD and CARD domain containing	− 3.54
Hs. 109755	NM_003821	RIPK2	Receptor-interacting serine-threonine kinase 2	4.39
Hs. 241570	NM_000594	TNF	Tumor necrosis factor	2.76
Hs. 661668	NM_003842	TNFRSF10B	Tumor necrosis factor receptor superfamily, member 10b	2.47
Hs. 256278	NM_001066	TNFRSF1B	Tumor necrosis factor receptor superfamily, member 1B	− 2.11
Hs. 738942	NM_001561	TNFRSF9	Tumor necrosis factor receptor superfamily, member 9	2.42
Hs. 478275	NM_003810	TNFSF10	Tumor necrosis factor (ligand) superfamily, member 10	− 4.02
Hs. 654445	NM_001244	TNFSF8	Tumor necrosis factor (ligand) superfamily , member 8	2.14
Hs. 460996	NM_003789	TRADD	TNFRSF1A-associated via death domain	− 2.09

QPCR array data corresponding to apoptotic genes expression profile. A total of 84 markers were analyzed following incubation with an intermediate concentration of eugenol estimated at 200 µM. The treatment duration was limited to 24 h. Unigene designator, Refseq identifier, symbol and description corresponding to all factors showing discrepancies relative to untreated control are summarized in Table 2. Only the genes displaying over two-fold variation were taken into consideration. This indicative study corresponds to only one experiment (n = 1).

### Eugenol limits Ca9-22 cell migration through MMP1/3 downregulation

**Figure 4:**
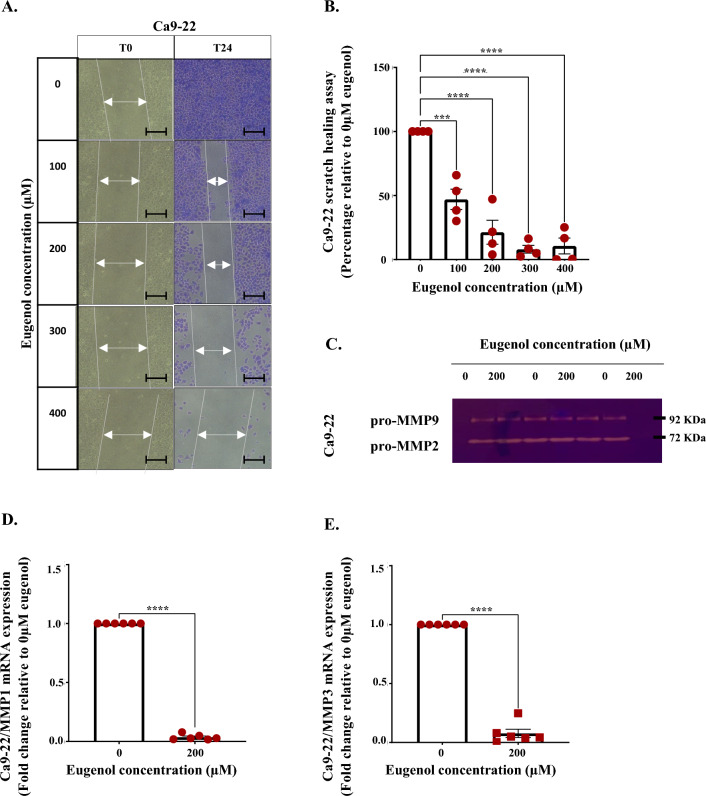
Effect of eugenol on Ca9-22 cells migration. (**A**) and (**B**) Tracing scratch healing capacities through estimation of scratch diameter alterations following administration of different eugenol concentrations. Figures were taken at the beginning of the experiment and after 24 h. Scale bars corresponds to 500 µm (n = 4). (**C**) pro-MMP2, pro-MMP9 (n = 3), (**D**) pro-MMP1 (n = 6) and (**E**) pro-MMP3 (n = 6) expression as per the gelatin zymography and QPCR assays. Presented data are expressed as mean values ± SEM of three to six independent experiments. ****p < 0.0001 were considered as statistically significant.

Scratch assay data analysis demonstrated that eugenol limited Ca9-22 cells migration and thus healing in a dose dependent manner. Scratch closure is totally marked in the control, whereas following stimulation with 100 µM eugenol, an inhibition of Ca9-22 migration potential by 53% was recorded, in the absence of any significant impact on cytotoxicity as well as on cell proliferation (Fig. 4A and B). To understand the underlying mechanisms, we then focused on the members of several secreted protease groups including the MMP1 collagenase, the MMP2/9 gelatinases and the MMP3 stromelysin given the diversity of the substrate range^43^. As per the gelatin zymography and QPCR results, it appears that this effect is not mediated through MMP2 and MMP9 modulation (Fig. 4C and Supplementary Fig. 5) but rather associated to MMP1 and MMP3 elimination (Fig. 4D and E). Even though eugenol exhibited the same impact on SCC-9 migration, the causal processes were shown to be different and inactive MMPs complex formation is detected. No effect at the level of MMP1, MMP2 and MMP9 expression was recorded. Only MMP3 upregulation was observed following treatment with 300 µM eugenol (IC_50_ for SCC9) (Supplementary Fig. 4 and 5).

### Eugenol effects are mediated by pSTAT5, pP38, pNF-kB modulation

**Figure 5:**
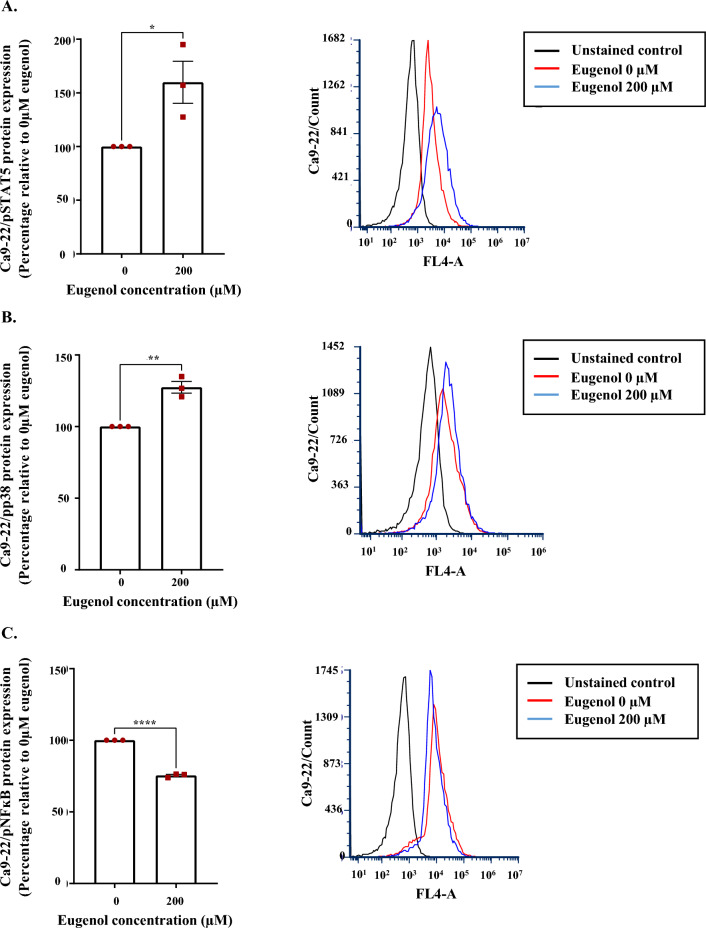
Effect of eugenol on gingival carcinoma signaling pathways. Flow cytometry data showing phosphorylation rates of (**A**) STAT5 (n = 3), (**B**) NF-ĸB (n = 3) and (**C**) P38 (n = 3) signaling molecules following incubation with an intermediate eugenol concentration estimated at 200 µM. All presented data are expressed as mean values ± SEM of three independent experiments. *p < 0.05, **p < 0.01, and ****p < 0.0001 were considered as statistically significant.

Flow cytometry results showed that supplementation with 200 µM eugenol for 24 h stimulated the phosphorylation of STAT5 (Fig. 5A) and P38 (Fig. 5B). Phospho-NF-ĸB levels on the other hand were negatively regulated (Fig. 5C). A slight effect was detected at the level of pSTAT1 and pERK1/2 (data not shown). It seems that gingival and tongue carcinoma modulate distinct signaling molecules as no effect was shown on the phosphorylated forms of the aforementioned signaling molecules following incubation with 300 µM eugenol. Only a miniscule increase of STAT3 phosphorylation was discovered (data not shown).

## Discussion

Despite current use in dental clinics for its antibacterial effect, eugenol implication as an alternative treatment option for cancer is beginning to gain ground in research. In fact, eugenol effectiveness towards various cancer models including melanoma, leukemia and skin tumors was previously validated^32^. However, few studies have been carried out to validate its effects on oral cancer, and even less is available on gingival carcinoma, one of the common types of oral cancers. Although Teho Koh et al*.* failed to demonstrate the potent tumor specific effect of eugenol dental compound against human oral squamous cell carcinoma cell lines HSC-2, HSC-4 and Ca9-22 and this is relative to normal cells and other anticancer drugs^44^, Surducan et al*.* were able to report inhibition of SCC-4 tongue carcinoma proliferation in a dose dependant manner. IC50 was registered around 750 µM and a high concentration, in the order of 500 µM, was required to induce apoptosis^38^. In another paper, eugenol decreased colony formation and around 900 µM was needed to eliminate half of the SCC-9 cells^37^. This was to be expected as tongue carcinoma is generally regarded as a more aggressive and a highly metastatic entity as opposed to other oral subsites^45^. For instance, cervical lymph node metastasis, greatly correlated with tongue tumors, is known to reduce survival chances half^6,8,46^. In the present study, we focused on eugenol effectiveness against a less aggressive form occurring in the gingival tissue. Our results showed that eugenol inhibited Ca9-22 oral cancer cells proliferation, and that the effect registered was more remarkable as compared to tongue carcinoma. Tumor specific IC50s were recorded around 200 µM for Ca9-22 and 300 µM for the SCC-9 cell line. Being selective to the Ca9-22 cancer cells, this paper is encouraging the potential use of eugenol for the specific management of gingival carcinoma and is aiming to decipher its mechanisms of action.

Given that eugenol was proven to be capable of inducing cytotoxicity and hindering cell proliferation and colony formation, it was imperative to test whether the observed outcomes were mediated by a direct impact on the progression of the cell cycle. Interestingly, we were able to confirm a blockade of Ca9-22 cells at the level of G0–G1 following supplementation of eugenol. Based on our QPCR array data, the tongue carcinoma cell line reacted in a different way. At this level, Saranya Varadarajan et al*.* showed that Cinnamomum verum J. Presl extract (bark) and its active constituents including eugenol demonstrated anticancer effects in vitro via apoptosis induction and S-phase arrest of the SCC-25 tongue carcinoma cell line^39^. Considering the novelty of the subject, it was important to focus on analyzing the determinants of Ca9-22 response to eugenol. Based on the western blot and QPCR array data, we were able to unravel key G1 phase markers, S phase components, DNA repair agents and controllers of G1/S transition implicated at this level. Primarily, we were able to show reduced expression of the cyclin dependent kinases (CDK) 4/6 and of their allosteric activator cyclin D1. Being a driver of G1/S transition, cyclin D1-CDK4/6 complex depletion, is likely to be implicated in the G0–G1 phase arrest. Generally speaking, eugenol mediated mitigation of CDK4/6 is expected to permit continuous retention of E2Fs transcription factors by the retinoblastoma tumor suppressor (RB) thus refraining cell cycle progression^47^. Besides, shortening in TFDP1 gene, a heterodimeric partner of activator E2F, will almost certainly inhibit DNA binding and activation of target genes^48^. Even though the RB family members are primarily known for inhibiting transition to the S phase, ablation of the cell cycle repressor RBL2 (Retinoblastoma-like 2) might be related to its antiapoptotic role^49,50^. This example illustrates the connection between the cell cycle and cell death^51^.

Contrary to cyclin D1, cyclin D2 upregulation by the effect of eugenol may contribute to induction and/or maintenance of a non-proliferative state, possibly through nuclear sequestration of the CDK2 catalytic subunit^52^, the expression of which was found to be downregulated. This is consistent with the outcome observed on cyclin E1 and can be explained by the fact that CDK2/E-type cyclins complex is not required due to obstruction of G1/S transition^53^. Of note, cyclin D2 is also expected to be associated with CDK4 depletion during growth arrest^52^. On the other hand, cyclin-dependent kinase 5 regulatory subunit associated protein 1 (CDK5RAP1) inhibition by eugenol reflect maintenance of the CDK5 kinase activity. The later is known to favor apoptosis and to block the cell cycle at the level of the G0/G1 phase through direct interaction and sequestration of cyclin D1 and E2F1 in the nucleus^54,55^. Beside the phosphorylation status and the cyclin partner availability, CDK activity is further modulated by the increase in p15^INK4B^, p21^CDKN1A^ and p27^CDKN1B^^56^ CDK inhibitory proteins (CDKI)^57^. Furthermore, it appears that SCF^SKP2^ ubiquitin ligase component SKP2 downregulation in response to eugenol limited the degradation of p21 and p27 and thus loss of oncogenesis control At this level, the NUCKS1-SKP2-p21/p27 pathway is recognized as a checkpoint for G1/S transition^58^.

The modified factors also corresponded to key S phase components where eugenol is thought to restrict MCM2–7 complex formation and eventually DNA binding and replication obstruction^59^. Besides replication promotors, we noticed alterations in two major cell cycle checkpoints: The ATM/CHK2 and the ATR/CHK1^60,61^ following eugenol supplementation. In fact, CHEK2 downregulation together with its downstream effectors BRCA2^62^ signal a preference towards apoptosis rather than DNA repair and subsequent cell cycle progression. Furthermore, upregulation of the RAD1 component of the RAD9/RAD1/HUS1 complex doesn’t seem sufficient to induce ATR-dependent CHK1 activation and repair despite the damage inflicted to the cells^63^. Cell cycle checkpoint ATM and ATR are reported to control directly several members of the MCM complex to prevent genomic damage^64^. Cyclin F CDK, reduced by eugenol, is know to be capable of initiating checkpoint response in G2^65^ and to ensure mitosis fidelity through control of centrosome duplication^66^. Dual roles have been described for cyclin F^67,68^, and our results follow the example of the ovarian cancer where cyclin F enhanced proliferation and invasion of cancer cells and was linked with a poor prognosis in patients^68^. Another extensively described checkpoint process downregulated by eugenol involves the APC2 (also known as ANAPC2) catalytic subunit of the anaphase promoting complex (APC/C). APC, reigning during the anaphase to the end of the G1 phase, guarantees proper cell growth and division through modulation of multiple processes including chromosome segregation. Following the example of acute myeloid leukemia, we encourage exploring the potential of APC/C as a molecular biomarker for oral cancer prognosis^69^.

Being well documented in the literature, the possible effect of eugenol on apoptosis was explored^32^. As per our results, it appears that cell death was imposed through PARP1 modulation. In fact, protein poly(ADP-ribosyl)ation (PARylation) by poly(ADP-ribose) polymerase 1 (PARP1) is implicated in DNA repair among other intracellular processes. More specifically, PARP1 suppression leads to accumulation of DNA errors and sensitizes cancer cells to death in the presence of conventional anti-cancer drugs options. All of this encourages the potential use of eugenol-cisplatin combinations for the management of cancer^70,71^. On the other hand, the pro-apoptotic BCL-2 family member Noxa is strongly inhibited by the action of eugenol. Given that Noxa depletion is a common mechanism of drug resistance, better outcomes in the form of complete tumor regression and durable remission, might require overcoming Noxa destabilisation^72^. Based on the QPCR array data, death induction by eugenol is credited to multiple other proteins implicated in pyroptosis as well as in the extrinsic and intrinsic apoptotic pathways. As for the extrinsic apoptotic pathway, death receptors activation (Fas, TNFRSF10B, TNFRSF9), death ligands availability (TNF, TNFSF8) and apoptotic initiators activation (caspase 10) are the determinants of eugenol action at this level. Regarding the intrinsic mitochondrial pathway, this accounts mainly on the translational upregulation of the initiator caspase 9, DIABLO and BCL2L11/Bim^73,74^. Furthermore, an inflammatory form of apoptosis, seems to be heavily targeted by eugenol. More closely, 15.61 folds increase at the level of the inflammatory caspase 5 signal inflammasomes assembly and pyroptosis induction^75^ thus enabling cell perforation as well as cytokine maturation and release^76^. Pyroptosis is known to be triggered by other chemotherapy agents and natural compounds^76^, including cisplatin^77^. Attention directed towards eugenol mediated inflammation could be of a great interest to understand drug functional characteristics.

From another angle, eugenol capacity to hinder invasion merits special attention due to promising attenuation of disease aggressivity. In our study, we were able to show that eugenol limited cancer cell migration probably due to altered matrix metalloproteinases (MMPs) secretion. More specifically, decreased MMP1 collagenase and MMP3 stromelysin levels were detected in Ca9-22 cells. At this level, a reduction in extracellular matrix (ECMs) degradation and modulation of cell behavior and cell biophysical properties relevant to invasion by means of integrins, focal adhesion kinase^78^ and cell contractility modulation^79^ should be considered. As for SCC-9, MMP3 upregulation and formation of MMPs complexes lacking proteolytic activity are expected to be the causal agents for tongue cancer cells migration restriction. The results at the level of MMP3 are conflicting and can be explained by the fact that this specific protease behavior is context dependent. For instance, MMP3 favors breast cancer progression while being a protective agent promoting leukocytes recruitment in skin carcinogenesis^80^. These results are particularly encouraging for exploring eugenol impact on the angiogenesis process as elastolytic MMP3 are known to generate biologically functional angiogenesis inhibitor, angiostatin^81^. On the other hand, high molecular weight MMPs complex (130, 170, and 220 kDa) formation ensure stable sequestration of MMP2, MMP9, TIMP1 (tissue inhibitors of metalloproteinases), and enzyme stabilizer NGAL (neutrophil gelatinase-associated lipocalin)^82^. Given that the MMP complexes are detected in biological fluids, further investigation is required for their adoption as a tongue carcinoma diagnosis signature^83^.

Our last aim was to explore the cell signaling molecules carrying the message instructed by eugenol. Our results showed upregulation of the STAT5 and p38 phosphorylation levels as opposed to NF-κB. STAT5 tumor suppressor activities were previously demonstrated in breast cancer and chronic liver disease patients. This is in line with a study showing that loss of STAT5 in mouse embryonic fibroblasts and hepatocytes reduced the expression of the CDKIs p15^INK4b^ and p21^CIP^ thus leading to enhanced cell cycle progression. Other anti-oncogenes including SOCS1, p53 and PML were also shown to be activated by STAT5 in the context of differentiation and senescence regulation^84^. Likewise, the tumor suppressor role of the p38 MAPK, originally activated by environmental and genotoxic stresses, might derive from its capacity to induce dormancy, promote apoptosis, block differentiation and suppress metastasis. In this respect, it should be pointed out that tumor requirements are completely stage-dependent and that more advanced tumor stages can benefit from high p38 levels^85^. Quite on the contrary, aberrant NF-κB activation is associated with multidrug resistance and is identified as a tumor signature that drives apoptosis evasion, disease recurrence and therapy resistance in both solid and haematological malignancies. In the context of the life/death balance, proliferation, migration, autophagy and necroptosis processes are also revealed to be under the influence of the NF-κB pathway^86,87^. However, considering that all the aforementioned signaling messengers can act as either malignant promotors or tumor suppressors according to the circumstances^85,88,89^, treatments should focus on balance restoration rather than complete activation or inhibition of certain signaling molecules^84^. Due to the unveiled complexity, attention is to be directed towards the eugenol natural compound.

As per the global cancer observatory, it is predicted that the incidence and mortality corresponding to oral squamous cell carcinoma will score an increase by up to 40% in 2040^90^ with an estimated elevation in female patients affected^91^ thereby urging more efforts at the level of disease prevention and proper management. Following the examples available in literature, our study provides a solid ground to go and assess eugenol efficacy, whether alone or in combination with chemotherapy, in animal models^35,36^ and raises multiple questions regarding introduction to clinical settings. Although considered safe at a dose of 2.5 mg/kg body weight and commercially available for multiple applications such as food flavoring since 1940, clinical trials evaluating its potential for the management of oral cancer has not been launched for the moment^28^. Multiple factors should be taken into consideration before administering the eugenol drug in humans. This mainly includes the routes of administration, dose selection, drug interactions and patient population^92^.

Based on the fact that sequential administration of eugenol is proposed^35^ mainly in the event of chemotherapy drug resistance^36^, repetitive intravenous administration might be overwhelming for patients. Thus, oral, and topical routes can be considered. At these levels, multiple scenarios can be adopted based on available literature data. For instance, modeling after the black raspberry mucoadhesive gel success^93^, topical application of eugenol-based paste is rather suggested for premalignant lesions management. This is attributed to limited cancer site accessibility. It is noteworthy that eugenol paste demonstrated tolerability and effectiveness in preventing alveolar osteitis as well as in supporting wound healing in 270 patients having their third molar extracted^94^. Dissolvable troches can also be an option as they ensure prolonged oral mucosa contact time. In this context, one paper reported that black raspberries administration in the form of troche allowed successful targeting oral squamous cells carcinoma tissues and reduced antiapoptotic and proinflammatory molecular biomarkers^95^. However, following oral administration of gelatin capsules containing eugenol in male and female healthy volunteers, eugenol bioavailability was questioned as it was shown to rapidly peak in blood before being metabolized and almost entirely excreted in the urine after 24 h^29,30^. Other issues to be considered are its low solubility in water, physiological barriers and targeted delivery of high drug concentrations to cancer sites. To tackle these challenges, delivery systems including liposomes, nanoparticles and phospholipid complexes can be implemented^96,97^. For instance, hydrogels containing eugenol-loaded solid lipid nanoparticles improved delivery by at least sixfold to fungi infected cells^98^. Enhancing thermal stability of inclusion complexes may also ensure slow release of eugenol^28^. Finally, customizing treatment according to patients’ needs and offering precise therapies, mainly in terms of personalized dosage, is paramount to enhance therapeutic effectiveness based on the differences observed between the gingival and tongue squamous carcinoma cell lines^99^.

## Conclusion

Eugenol effectiveness covers gingival carcinoma following the example of other cancer variants. Our drug was also shown to be tumor-selective, and its therapeutic potential was declared inconsistent relative to oral cancer subtypes with tongue carcinoma being more resistant. In summary, eugenol use as an adjuvant treatment option, either alone or in combination with chemotherapy, merits further investigation for the management of gingival carcinoma in patients with advanced disease state.

### Supplementary Information

Supplementary Figure 1.

Supplementary Figure 2.

Supplementary Figure 3.

Supplementary Figure 4.

Supplementary Figure 5.

Supplementary Table 1.

Supplementary Table 2.

## Data Availability

All data generated or analysed during this study are included in this published article and its supplementary information files.

## Abbreviations

AnxV: Annexin V
APC/C: Anaphase promoting complex
Ca9-22: Human gingival carcinoma cell line
CDK: Cyclin dependent kinase
CDK5RAP1: Cyclin-dependent kinase 5 regulatory subunit associated protein 1
CDKI: Cyclin dependent kinases inhibitors
F: Fungizone
FBS: Fetal bovine serum
GMSM-K: Non-oncogenic human oral epithelial cell line
LDH: Lactate dehydrogenase
MMPs: Matrix metalloproteinases
MTT: 3-(4,5-Dimethylthiazol-2-yl)-2,5-diphenyltetrazolium bromide
PARP1: Poly(ADP-ribose) polymerase 1
PI: Propidium iodide
P/S: Penicillin/streptomycin
RBL2: Retinoblastoma-like 2
RB: Retinoblastoma tumor suppressor
SCC-9: Tongue carcinoma cell line

## References

[1] Warnakulasuriya S, Kerr AR (2021). Oral cancer screening: Past, present, and future. J. Dent. Res..

[2] Bagan J, Sarrion G, Jimenez Y (2010). Oral cancer: Clinical features. Oral Oncol..

[3] Chamoli A (2021). Overview of oral cavity squamous cell carcinoma: Risk factors, mechanisms, and diagnostics. Oral Oncol..

[4] Maier H, Zöller J, Herrmann A, Kreiss M, Heller WD (1993). Dental status and oral hygiene in patients with head and neck cancer. Otolaryngol. Head Neck Surg. Off. J. Am. Acad. Otolaryngol. Head Neck Surg..

[5] Taghavi N, Yazdi I (2007). Type of food and risk of oral cancer. Arch. Iran. Med..

[6] Review on applications of metastatic lymph node based radiomic assessment in nasopharyngeal carcinoma. *J. Cancer Metastasis Treat.* (2023). 10.20517/2394-4722.2022.100.

[7] Pfister DG (2020). Head and Neck Cancers, Version 2.2020, NCCN clinical practice guidelines in oncology. J. Natl. Compr. Cancer Netw. JNCCN.

[8] Bußmann L (2020). Comparative effectiveness trial of transoral head and neck surgery followed by adjuvant radio(chemo)therapy versus primary radiochemotherapy for oropharyngeal cancer (TopROC). BMC Cancer.

[9] Cheraghlou S, Schettino A, Zogg CK, Judson BL (2018). Changing prognosis of oral cancer: An analysis of survival and treatment between 1973 and 2014. The Laryngoscope.

[10] Montero PH, Patel SG (2015). Cancer of the oral cavity. Surg. Oncol. Clin. N. Am..

[11] Dasari S, Bernard Tchounwou P (2014). Cisplatin in cancer therapy: Molecular mechanisms of action. Eur. J. Pharmacol..

[12] Cheng Y, Li S, Gao L, Zhi K, Ren W (2021). The molecular basis and therapeutic aspects of cisplatin resistance in oral squamous cell carcinoma. Front. Oncol..

[13] Semlali A, Beji S, Ajala I, Al-Zharani M, Rouabhia M (2023). Synergistic effects of new curcumin analog (PAC) and cisplatin on oral cancer therapy. Curr. Issues Mol. Biol..

[14] Nussbaumer S, Bonnabry P, Veuthey J-L, Fleury-Souverain S (2011). Analysis of anticancer drugs: A review. Talanta.

[15] Semlali A, Ajala I, Beji S, Al-Zharani MM, Rouabhia M (2023). Synergistic effect of anethole and platinum drug cisplatin against oral cancer cell growth and migration by inhibiting MAPKase, beta-catenin, and NF-κB pathways. Pharm. Basel Switz..

[16] Newman DJ, Cragg GM (2016). Natural products as sources of new drugs from 1981 to 2014. J. Nat. Prod..

[17] George BP, Chandran R, Abrahamse H (2021). Role of phytochemicals in cancer chemoprevention: Insights. Antioxid. Basel Switz..

[18] Sikdar S, Mukherjee A, Ghosh S, Khuda-Bukhsh AR (2014). Condurango glycoside-rich components stimulate DNA damage-induced cell cycle arrest and ROS-mediated caspase-3 dependent apoptosis through inhibition of cell-proliferation in lung cancer, in vitro and in vivo. Environ. Toxicol. Pharmacol..

[19] Sha J, Bai Y, Ngo HX, Okui T, Kanno T (2021). Overview of evidence-based chemotherapy for oral cancer: Focus on drug resistance related to the epithelial-mesenchymal transition. Biomolecules.

[20] Ayaz M (2022). Underlying anticancer mechanisms and synergistic combinations of phytochemicals with cancer chemotherapeutics: Potential benefits and risks. J. Food Qual..

[21] Choudhari AS, Mandave PC, Deshpande M, Ranjekar P, Prakash O (2019). Phytochemicals in cancer treatment: From preclinical studies to clinical practice. Front. Pharmacol..

[22] Semlali A, Contant C, Al-Otaibi B, Al-Jammaz I, Chandad F (2021). The curcumin analog (PAC) suppressed cell survival and induced apoptosis and autophagy in oral cancer cells. Sci. Rep..

[23] Contant C, Rouabhia M, Loubaki L, Chandad F, Semlali A (2021). Anethole induces anti-oral cancer activity by triggering apoptosis, autophagy and oxidative stress and by modulation of multiple signaling pathways. Sci. Rep..

[24] Semlali A, Beji S, Ajala I, Rouabhia M (2021). Effects of tetrahydrocannabinols on human oral cancer cell proliferation, apoptosis, autophagy, oxidative stress, and DNA damage. Arch. Oral Biol..

[25] Loubaki L, Rouabhia M, Zahrani MA, Amri AA, Semlali A (2022). Oxidative stress and autophagy mediate anti-cancer properties of cannabis derivatives in human oral cancer cells. Cancers.

[26] Nisar MF (2021). Pharmacological properties and health benefits of eugenol: A comprehensive review. Oxid. Med. Cell. Longev..

[27] Sarkic A, Stappen I (2018). Essential oils and their single compounds in cosmetics—A critical review. Cosmetics.

[28] Ulanowska M, Olas B (2021). Biological properties and prospects for the application of eugenol-a review. Int. J. Mol. Sci..

[29] Fischer IU, von Unruh GE, Dengler HJ (1990). The metabolism of eugenol in man. Xenobiotica Fate Foreign Compd. Biol. Syst..

[30] Guénette SA, Ross A, Marier J-F, Beaudry F, Vachon P (2007). Pharmacokinetics of eugenol and its effects on thermal hypersensitivity in rats. Eur. J. Pharmacol..

[31] Begum SN, Ray AS, Rahaman CH (2022). A comprehensive and systematic review on potential anticancer activities of eugenol: From pre-clinical evidence to molecular mechanisms of action. Phytomedicine Int. J. Phytother. Phytopharm..

[32] Jaganathan SK, Supriyanto E (2012). Antiproliferative and molecular mechanism of eugenol-induced apoptosis in cancer cells. Molecules.

[33] Mohamed AA, Alotaibi BM (2023). Essential oils of some medicinal plants and their biological activities: A mini review. J. Umm Al-Qura Univ. Appl. Sci..

[34] Zari AT, Zari TA, Hakeem KR (2021). Anticancer properties of eugenol: A review. Mol. Basel Switz..

[35] Islam SS, Aboussekhra A (2019). Sequential combination of cisplatin with eugenol targets ovarian cancer stem cells through the Notch-Hes1 signalling pathway. J. Exp. Clin. Cancer Res. CR.

[36] Islam SS (2018). Eugenol potentiates cisplatin anti-cancer activity through inhibition of ALDH-positive breast cancer stem cells and the NF-κB signaling pathway. Mol. Carcinog..

[37] Duan Y, Huang X, Qiao B, Ma R, Li J (2022). Eugenol inhibits the biological activities of an oral squamous cell carcinoma cell line SCC9 via targeting MIF. Anticancer Agents Med. Chem..

[38] Surducan D-A (2022). Eugenol induces apoptosis in tongue squamous carcinoma cells by mediating the expression of Bcl-2 family. Life Basel Switz..

[39] Varadarajan S, Narasimhan M, Balaji TM, Chamundeeswari DP, Sakthisekaran D (2020). In vitro anticancer effects of Cinnamomum verum J. Presl, cinnamaldehyde, 4 hydroxycinnamic acid and eugenol on an oral squamous cell carcinoma cell line. J. Contemp. Dent. Pract..

[40] Koh T (2013). Changes of metabolic profiles in an oral squamous cell carcinoma cell line induced by eugenol. Vivo Athens Greece.

[41] Gilchrist EP, Moyer MP, Shillitoe EJ, Clare N, Murrah VA (2000). Establishment of a human polyclonal oral epithelial cell line. Oral Surg. Oral Med. Oral Pathol. Oral Radiol. Endod..

[42] Overton WR (1988). Modified histogram subtraction technique for analysis of flow cytometry data. Cytometry.

[43] Cathcart J, Pulkoski-Gross A, Cao J (2015). Targeting matrix metalloproteinases in cancer: Bringing new life to old ideas. Genes Dis..

[44] Koh T, Machino M, Murakami Y, Umemura N, Sakagami H (2013). Cytotoxicity of dental compounds towards human oral squamous cell carcinoma and normal oral cells. Vivo Athens Greece.

[45] Bello IO, Soini Y, Salo T (2010). Prognostic evaluation of oral tongue cancer: Means, markers and perspectives (II). Oral Oncol..

[46] López F (2016). Cervical lymph node metastases from remote primary tumor sites. Head Neck.

[47] Wang B (2021). Breast cancer resistance to cyclin-dependent kinases 4/6 inhibitors: Intricacy of the molecular mechanisms. Front. Oncol..

[48] Nakajima R (2023). The TFDP1 gene coding for DP1, the heterodimeric partner of the transcription factor E2F, is a target of deregulated E2F. Biochem. Biophys. Res. Commun..

[49] Chen J, Xia P, Liu Y, Kogan C, Cheng Z (2022). Loss of Rbl2 (Retinoblastoma-Like 2) exacerbates myocardial ischemia/reperfusion injury. J. Am. Heart Assoc..

[50] Xia P (2023). RBL2 regulates cardiac sensitivity to anthracycline chemotherapy. JACC CardioOncol..

[51] King KL, Cidlowski JA (1995). Cell cycle and apoptosis: Common pathways to life and death. J. Cell. Biochem..

[52] Meyyappan M, Wong H, Hull C, Riabowol KT (1998). Increased expression of cyclin D2 during multiple states of growth arrest in primary and established cells. Mol. Cell. Biol..

[53] Fagundes R, Teixeira LK (2021). Cyclin E/CDK2: DNA replication, replication stress and genomic instability. Front. Cell Dev. Biol..

[54] Zhang J, Herrup K (2008). Cdk5 and the non-catalytic arrest of the neuronal cell cycle. Cell Cycle Georget. Tex.

[55] Wang H, Wei L, Li C, Zhou J, Li Z (2015). CDK5RAP1 deficiency induces cell cycle arrest and apoptosis in human breast cancer cell line by the ROS/JNK signaling pathway. Oncol. Rep..

[56] Qie S, Diehl JA (2016). Cyclin D1, cancer progression, and opportunities in cancer treatment. J. Mol. Med..

[57] Karimian A, Ahmadi Y, Yousefi B (2016). Multiple functions of p21 in cell cycle, apoptosis and transcriptional regulation after DNA damage. DNA Repair.

[58] Hume S (2021). The NUCKS1-SKP2-p21/p27 axis controls S phase entry. Nat. Commun..

[59] Neves H, Kwok HF (2017). In sickness and in health: The many roles of the minichromosome maintenance proteins. Biochim. Biophys. Acta Rev. Cancer.

[60] Reinhardt HC, Yaffe MB (2009). Kinases that control the cell cycle in response to DNA damage: Chk1, Chk2, and MK2. Curr. Opin. Cell Biol..

[61] Bartek J, Lukas J (2003). Chk1 and Chk2 kinases in checkpoint control and cancer. Cancer Cell.

[62] Apostolou P, Papasotiriou I (2017). Current perspectives on CHEK2 mutations in breast cancer. Breast Cancer Dove Med. Press.

[63] Bao S (2004). Disruption of the Rad9/Rad1/Hus1 (9-1-1) complex leads to checkpoint signaling and replication defects. Oncogene.

[64] Cortez D, Glick G, Elledge SJ (2004). Minichromosome maintenance proteins are direct targets of the ATM and ATR checkpoint kinases. Proc. Natl. Acad. Sci. USA.

[65] Klein DK (2015). Cyclin F suppresses B-Myb activity to promote cell cycle checkpoint control. Nat. Commun..

[66] D’Angiolella V (2010). SCF(Cyclin F) controls centrosome homeostasis and mitotic fidelity through CP110 degradation. Nature.

[67] Krajewski A (2020). Cyclin F downregulation affects epithelial-mesenchymal transition increasing proliferation and migration of the A-375 melanoma cell line. Cancer Manag. Res..

[68] Li Y (2020). Cyclin F and KIF20A, FOXM1 target genes, increase proliferation and invasion of ovarian cancer cells. Exp. Cell Res..

[69] Rahimi H (2015). The expression pattern of APC2 and APC7 in various cancer cell lines and AML patients. Adv. Med. Sci..

[70] Tempka D (2018). Downregulation of PARP1 transcription by CDK4/6 inhibitors sensitizes human lung cancer cells to anticancer drug-induced death by impairing OGG1-dependent base excision repair. Redox Biol..

[71] Fam HK (2013). TDP1 and PARP1 deficiency are cytotoxic to rhabdomyosarcoma cells. Mol. Cancer Res. MCR.

[72] Montero J (2019). Destabilization of NOXA mRNA as a common resistance mechanism to targeted therapies. Nat. Commun..

[73] Wachmann K (2010). Activation and specificity of human caspase-10. Biochemistry.

[74] Fulda S, Debatin K-M (2006). Extrinsic versus intrinsic apoptosis pathways in anticancer chemotherapy. Oncogene.

[75] Martinon F, Tschopp J (2007). Inflammatory caspases and inflammasomes: Master switches of inflammation. Cell Death Differ..

[76] Lu L (2022). Emerging mechanisms of pyroptosis and its therapeutic strategy in cancer. Cell Death Discov..

[77] Wang S (2022). GSDME is related to prognosis and response to chemotherapy in oral cancer. J. Dent. Res..

[78] Das A, Monteiro M, Barai A, Kumar S, Sen S (2017). MMP proteolytic activity regulates cancer invasiveness by modulating integrins. Sci. Rep..

[79] Orgaz JL (2014). Diverse matrix metalloproteinase functions regulate cancer amoeboid migration. Nat. Commun..

[80] Frieling JS, Li T, Tauro M, Lynch CC (2020). Prostate cancer-derived MMP-3 controls intrinsic cell growth and extrinsic angiogenesis. Neoplasia NY N.

[81] Cornelius LA (1998). Matrix metalloproteinases generate angiostatin: Effects on neovascularization. J. Immunol. Baltim. Md.

[82] Kiczak L (2013). Expression and complex formation of MMP9, MMP2, NGAL, and TIMP1 in porcine myocardium but not in skeletal muscles in male pigs with tachycardia-induced systolic heart failure. BioMed Res. Int..

[83] Roy R (2008). Tumor-specific urinary matrix metalloproteinase fingerprinting: identification of high molecular weight urinary matrix metalloproteinase species. Clin. Cancer Res Off. J. Am. Assoc. Cancer Res..

[84] Ferbeyre G, Moriggl R (2011). The role of Stat5 transcription factors as tumor suppressors or oncogenes. Biochim. Biophys. Acta.

[85] Martínez-Limón A, Joaquin M, Caballero M, Posas F, de Nadal E (2020). The p38 pathway: From biology to cancer therapy. Int. J. Mol. Sci..

[86] Verzella D (2020). Life, death, and autophagy in cancer: NF-κB turns up everywhere. Cell Death Dis..

[87] Dolcet X, Llobet D, Pallares J, Matias-Guiu X (2005). NF-kB in development and progression of human cancer. Virchows Arch. Int. J. Pathol..

[88] Halim CE, Deng S, Ong MS, Yap CT (2020). Involvement of STAT5 in oncogenesis. Biomedicines.

[89] Kaltschmidt B (2000). The pro- or anti-apoptotic function of NF-kappaB is determined by the nature of the apoptotic stimulus. Eur. J. Biochem..

[90] Oral cancer—The fight must go on against all odds. *Evid. Based Dent.* **23**, 4–5 (2022). 10.1038/s41432-022-0243-1 35338315

[91] Infante-Cossio P, Duran-Romero A-J, Castaño-Seiquer A, Martinez-De-Fuentes R, Pereyra-Rodriguez J-J (2022). Estimated projection of oral cavity and oropharyngeal cancer deaths in Spain to 2044. BMC Oral Health.

[92] Shen J (2019). Design and conduct considerations for first-in-human trials. Clin. Transl. Sci..

[93] Mallery SR (2014). Topical application of a mucoadhesive freeze-dried black raspberry gel induces clinical and histologic regression and reduces loss of heterozygosity events in premalignant oral intraepithelial lesions: Results from a multicentered, placebo-controlled clinical trial. Off. J. Am. Assoc. Cancer Res..

[94] Jesudasan JS, Wahab PUA, Sekhar MRM (2015). Effectiveness of 0.2% chlorhexidine gel and a eugenol-based paste on postoperative alveolar osteitis in patients having third molars extracted: A randomised controlled clinical trial. Br. J. Oral Maxillofac. Surg..

[95] Knobloch TJ (2016). Suppression of proinflammatory and prosurvival biomarkers in oral cancer patients consuming a black raspberry phytochemical-rich troche. Cancer Prev. Res. Phila. Pa.

[96] Aburel OM (2021). Pleiotropic effects of eugenol: The good, the bad, and the unknown. Oxid. Med. Cell. Longev..

[97] Lee T-Y, Tseng Y-H (2020). The potential of phytochemicals in oral cancer prevention and therapy: A review of the evidence. Biomolecules.

[98] Garg A, Singh S (2014). Targeting of eugenol-loaded solid lipid nanoparticles to the epidermal layer of human skin. Nanomedicine.

[99] Puccetti M, Pariano M, Schoubben A, Giovagnoli S, Ricci M (2024). Biologics, theranostics, and personalized medicine in drug delivery systems. Pharmacol. Res..

