# Supplementary material for: Eugenol as a potential adjuvant therapy for gingival squamous cell carcinoma
Source: Sci Rep. 2024 May 13;14:10958. doi: 10.1038/s41598-024-60754-8 (PMC11091204; doi:10.1038/s41598-024-60754-8)
Supplement: Supplementary file 1 — Supplementary Figure 1. [file 41598_2024_60754_MOESM1_ESM.pptx]

## Slide 1
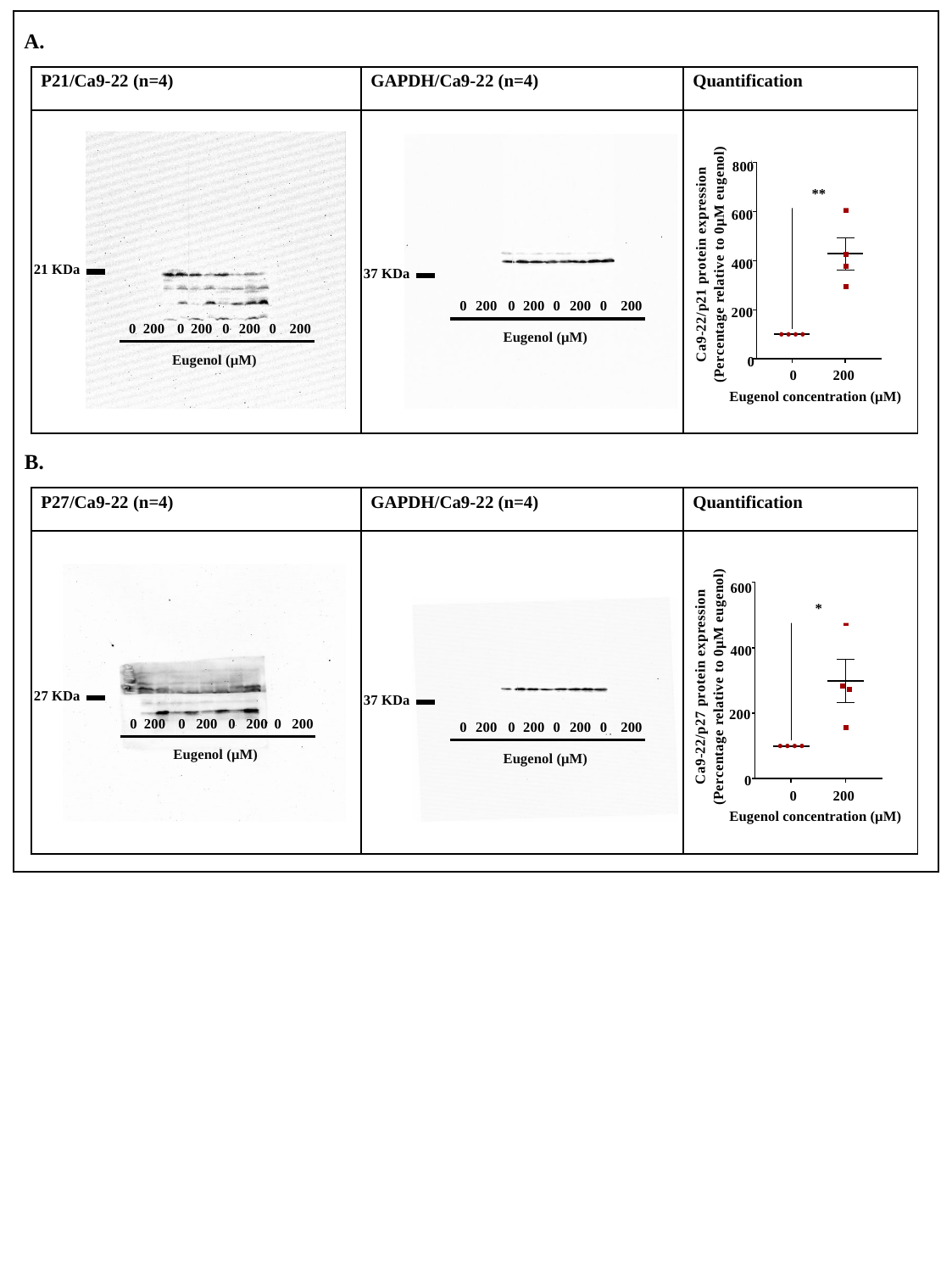

A.
| P21/Ca9-22 (n=4) | GAPDH/Ca9-22 (n=4) | Quantification |
| --- | --- | --- |
| | | |
800
**
600
Ca9-22/p21 protein expression
(Percentage relative to 0µM eugenol)
400
21 KDa
37 KDa
0
200
0
200
0
200
0
200
200
0
200
0
200
0
200
0
200
Eugenol (μM)
Eugenol (μM)
0
0
200
Eugenol concentration (µM)
B.
| P27/Ca9-22 (n=4) | GAPDH/Ca9-22 (n=4) | Quantification |
| --- | --- | --- |
| | | |
600
*
400
Ca9-22/p27 protein expression
(Percentage relative to 0µM eugenol)
27 KDa
37 KDa
200
0
200
0
200
0
200
0
200
0
200
0
200
0
200
0
200
Eugenol (μM)
Eugenol (μM)
0
0
200
Eugenol concentration (µM)
