# Supplementary material for: Eugenol as a potential adjuvant therapy for gingival squamous cell carcinoma
Source: Sci Rep. 2024 May 13;14:10958. doi: 10.1038/s41598-024-60754-8 (PMC11091204; doi:10.1038/s41598-024-60754-8)
Supplement: Supplementary file 2 — Supplementary Figure 2. [file 41598_2024_60754_MOESM2_ESM.pptx]

## Slide 1
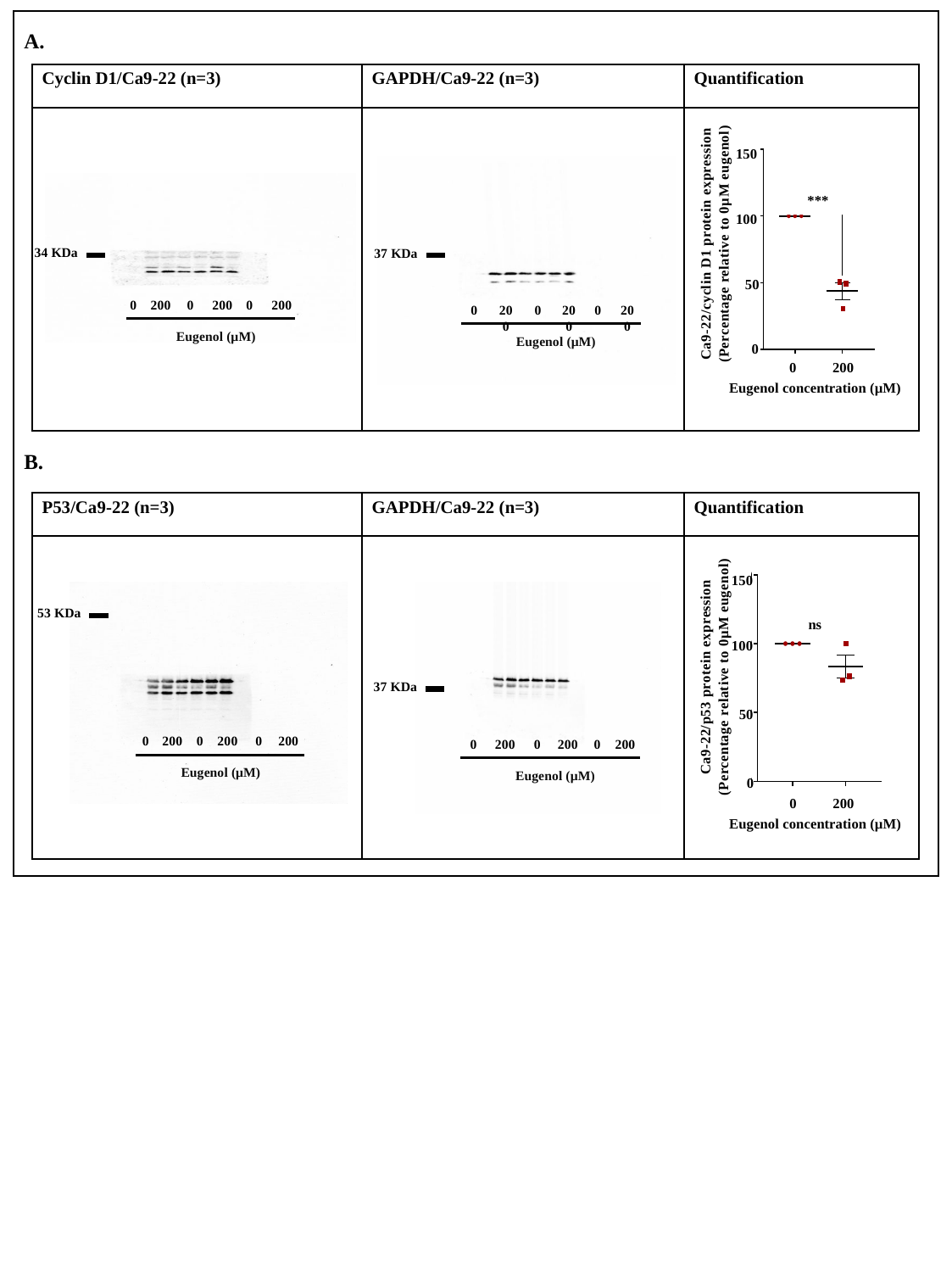

A.
| Cyclin D1/Ca9-22 (n=3) | GAPDH/Ca9-22 (n=3) | Quantification |
| --- | --- | --- |
| | | |
150
***
100
Ca9-22/cyclin D1 protein expression
(Percentage relative to 0µM eugenol)
34 KDa
37 KDa
50
0
200
0
200
0
200
0
200
0
200
0
200
Eugenol (μM)
Eugenol (μM)
0
0
200
Eugenol concentration (µM)
B.
| P53/Ca9-22 (n=3) | GAPDH/Ca9-22 (n=3) | Quantification |
| --- | --- | --- |
| | | |
150
53 KDa
ns
100
Ca9-22/p53 protein expression
(Percentage relative to 0µM eugenol)
37 KDa
50
0
200
0
200
0
200
0
200
0
200
0
200
Eugenol (μM)
Eugenol (μM)
0
0
200
Eugenol concentration (µM)
