# Supplementary material for: Eugenol as a potential adjuvant therapy for gingival squamous cell carcinoma
Source: Sci Rep. 2024 May 13;14:10958. doi: 10.1038/s41598-024-60754-8 (PMC11091204; doi:10.1038/s41598-024-60754-8)
Supplement: Supplementary file 4 — Supplementary Figure 4. [file 41598_2024_60754_MOESM4_ESM.pptx]

## Slide 1
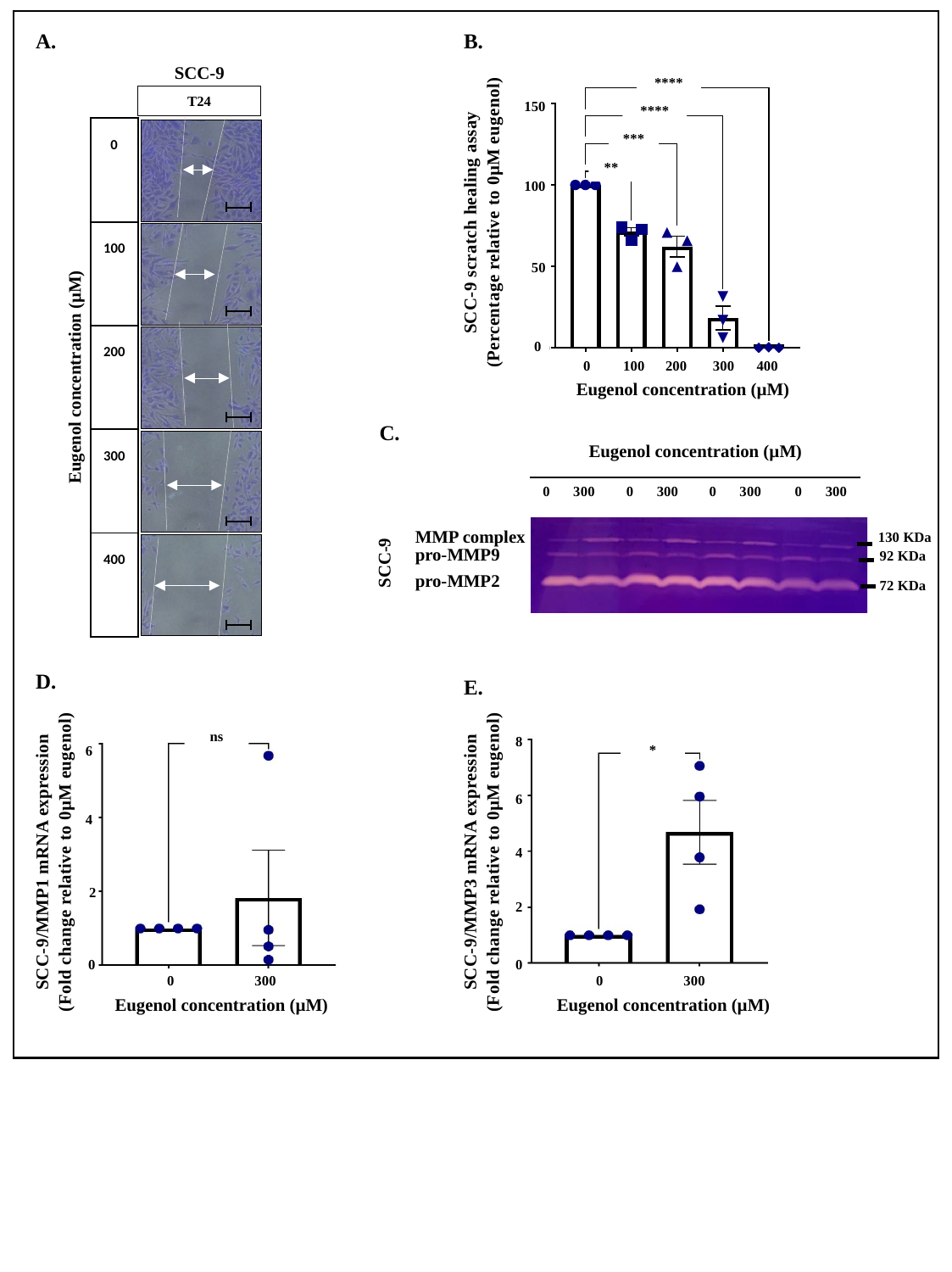

A.
B.
SCC-9
****
T24
150
****
| 0 |
| --- |
| 100 |
| 200 |
| 300 |
| 400 |
***
**
100
SCC-9 scratch healing assay
(Percentage relative to 0µM eugenol)
50
0
0
100
200
300
400
Eugenol concentration (µM)
Eugenol concentration (µM)
C.
Eugenol concentration (µM)
| 0 | 300 | 0 | 300 | 0 | 300 | 0 | 300 |
| --- | --- | --- | --- | --- | --- | --- | --- |
MMP complex
130 KDa
pro-MMP9
92 KDa
SCC-9
pro-MMP2
72 KDa
D.
E.
ns
8
*
6
6
4
SCC-9/MMP1 mRNA expression
(Fold change relative to 0µM eugenol)
SCC-9/MMP3 mRNA expression
(Fold change relative to 0µM eugenol)
4
2
2
0
0
0
300
0
300
Eugenol concentration (µM)
Eugenol concentration (µM)
