# Supplementary material for: Eugenol as a potential adjuvant therapy for gingival squamous cell carcinoma
Source: Sci Rep. 2024 May 13;14:10958. doi: 10.1038/s41598-024-60754-8 (PMC11091204; doi:10.1038/s41598-024-60754-8)
Supplement: Supplementary file 5 — Supplementary Figure 5. [file 41598_2024_60754_MOESM5_ESM.pptx]

## Slide 1
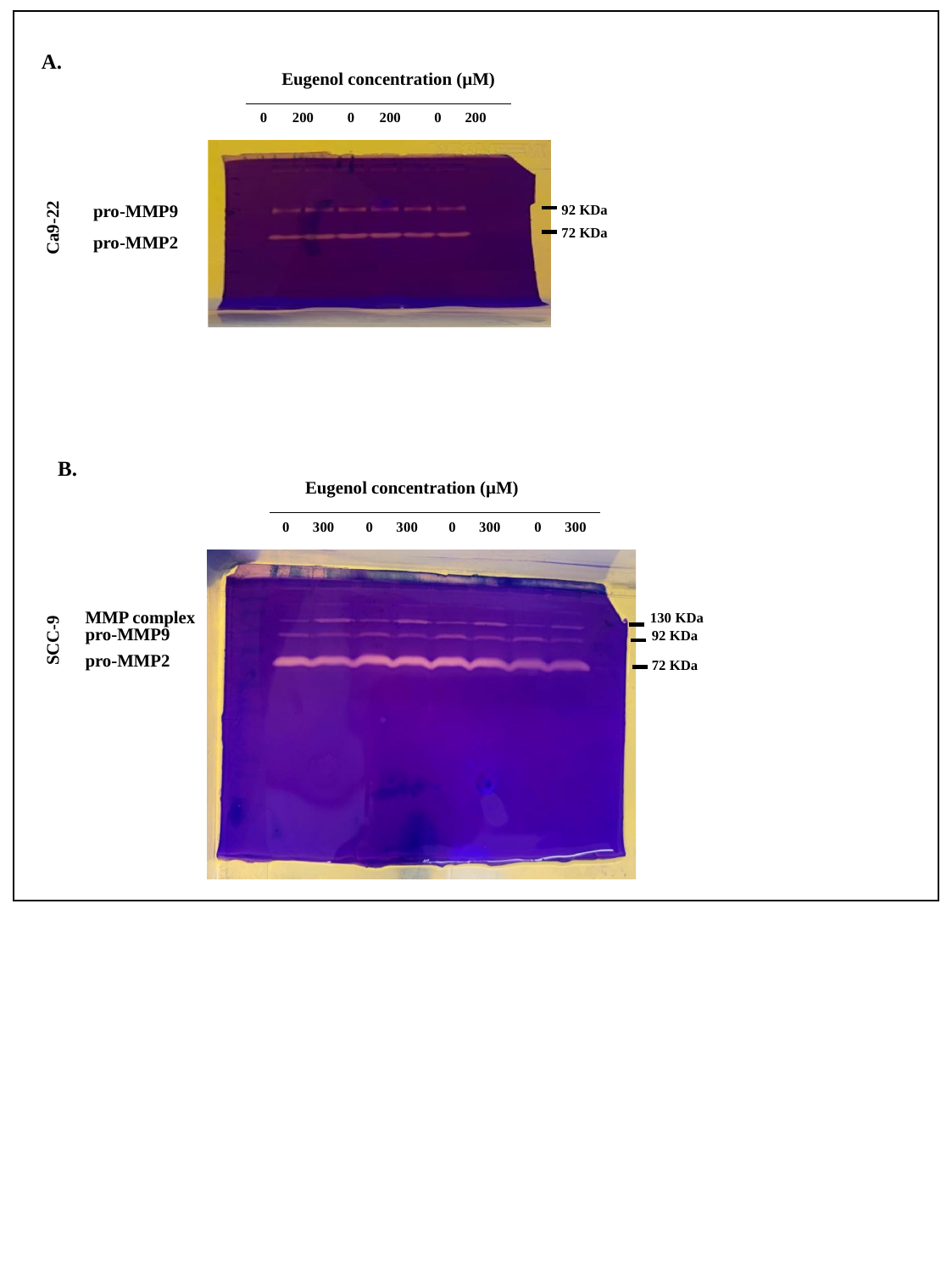

A.
Eugenol concentration (µM)
| 0 | 200 | 0 | 200 | 0 | 200 |
| --- | --- | --- | --- | --- | --- |
pro-MMP9
92 KDa
Ca9-22
72 KDa
pro-MMP2
B.
Eugenol concentration (µM)
| 0 | 300 | 0 | 300 | 0 | 300 | 0 | 300 |
| --- | --- | --- | --- | --- | --- | --- | --- |
MMP complex
130 KDa
pro-MMP9
92 KDa
SCC-9
pro-MMP2
72 KDa
