# Supplementary material for: Eugenol as a potential adjuvant therapy for gingival squamous cell carcinoma
Source: Sci Rep. 2024 May 13;14:10958. doi: 10.1038/s41598-024-60754-8 (PMC11091204; doi:10.1038/s41598-024-60754-8)
Supplement: Supplementary file 6 — Supplementary Table 1. [file 41598_2024_60754_MOESM6_ESM.pptx]

## Slide 1
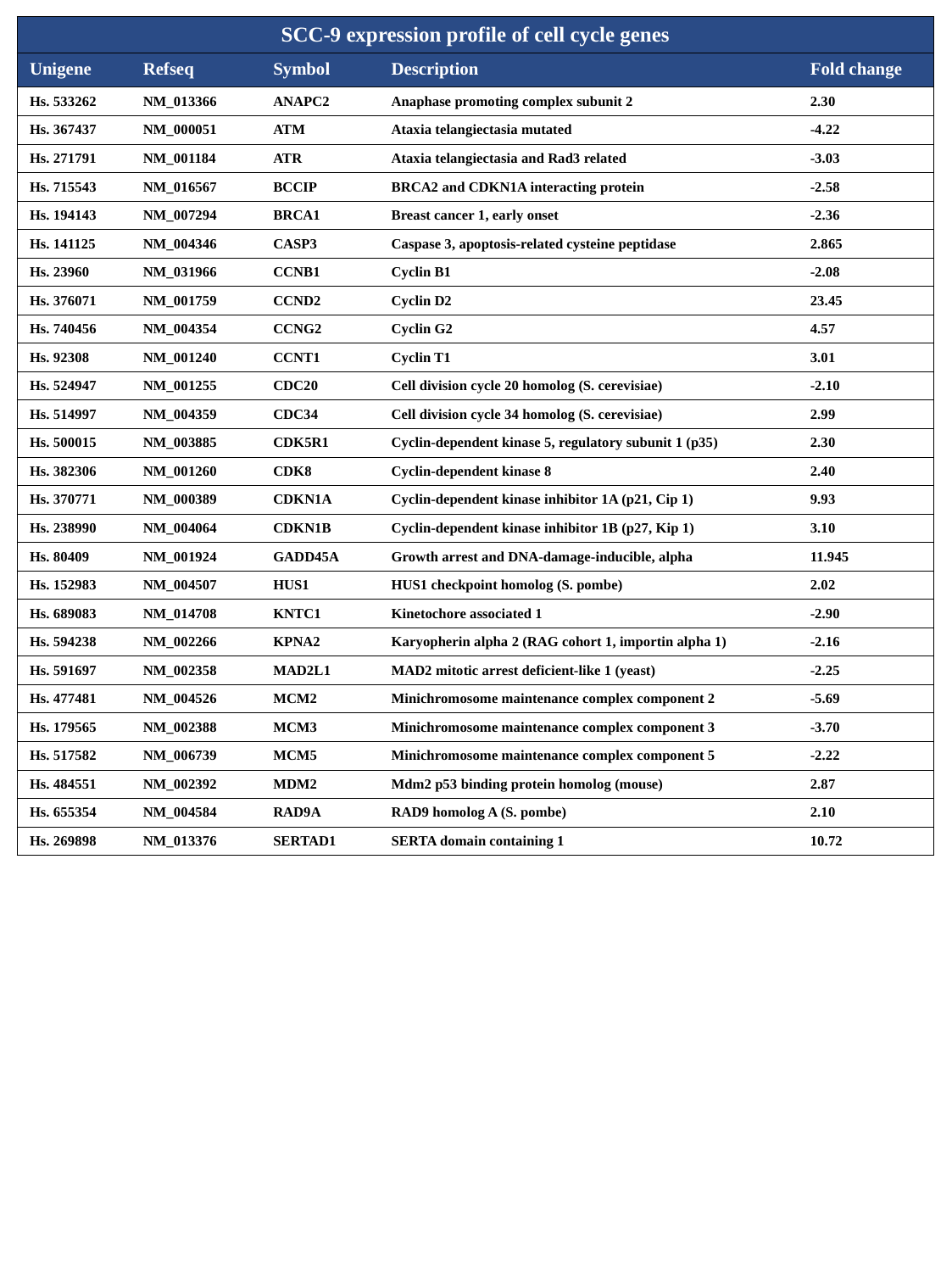

| SCC-9 expression profile of cell cycle genes | | | | |
| --- | --- | --- | --- | --- |
| Unigene | Refseq | Symbol | Description | Fold change |
| Hs. 533262 | NM\_013366 | ANAPC2 | Anaphase promoting complex subunit 2 | 2.30 |
| Hs. 367437 | NM\_000051 | ATM | Ataxia telangiectasia mutated | -4.22 |
| Hs. 271791 | NM\_001184 | ATR | Ataxia telangiectasia and Rad3 related | -3.03 |
| Hs. 715543 | NM\_016567 | BCCIP | BRCA2 and CDKN1A interacting protein | -2.58 |
| Hs. 194143 | NM\_007294 | BRCA1 | Breast cancer 1, early onset | -2.36 |
| Hs. 141125 | NM\_004346 | CASP3 | Caspase 3, apoptosis-related cysteine peptidase | 2.865 |
| Hs. 23960 | NM\_031966 | CCNB1 | Cyclin B1 | -2.08 |
| Hs. 376071 | NM\_001759 | CCND2 | Cyclin D2 | 23.45 |
| Hs. 740456 | NM\_004354 | CCNG2 | Cyclin G2 | 4.57 |
| Hs. 92308 | NM\_001240 | CCNT1 | Cyclin T1 | 3.01 |
| Hs. 524947 | NM\_001255 | CDC20 | Cell division cycle 20 homolog (S. cerevisiae) | -2.10 |
| Hs. 514997 | NM\_004359 | CDC34 | Cell division cycle 34 homolog (S. cerevisiae) | 2.99 |
| Hs. 500015 | NM\_003885 | CDK5R1 | Cyclin-dependent kinase 5, regulatory subunit 1 (p35) | 2.30 |
| Hs. 382306 | NM\_001260 | CDK8 | Cyclin-dependent kinase 8 | 2.40 |
| Hs. 370771 | NM\_000389 | CDKN1A | Cyclin-dependent kinase inhibitor 1A (p21, Cip 1) | 9.93 |
| Hs. 238990 | NM\_004064 | CDKN1B | Cyclin-dependent kinase inhibitor 1B (p27, Kip 1) | 3.10 |
| Hs. 80409 | NM\_001924 | GADD45A | Growth arrest and DNA-damage-inducible, alpha | 11.945 |
| Hs. 152983 | NM\_004507 | HUS1 | HUS1 checkpoint homolog (S. pombe) | 2.02 |
| Hs. 689083 | NM\_014708 | KNTC1 | Kinetochore associated 1 | -2.90 |
| Hs. 594238 | NM\_002266 | KPNA2 | Karyopherin alpha 2 (RAG cohort 1, importin alpha 1) | -2.16 |
| Hs. 591697 | NM\_002358 | MAD2L1 | MAD2 mitotic arrest deficient-like 1 (yeast) | -2.25 |
| Hs. 477481 | NM\_004526 | MCM2 | Minichromosome maintenance complex component 2 | -5.69 |
| Hs. 179565 | NM\_002388 | MCM3 | Minichromosome maintenance complex component 3 | -3.70 |
| Hs. 517582 | NM\_006739 | MCM5 | Minichromosome maintenance complex component 5 | -2.22 |
| Hs. 484551 | NM\_002392 | MDM2 | Mdm2 p53 binding protein homolog (mouse) | 2.87 |
| Hs. 655354 | NM\_004584 | RAD9A | RAD9 homolog A (S. pombe) | 2.10 |
| Hs. 269898 | NM\_013376 | SERTAD1 | SERTA domain containing 1 | 10.72 |
