# Supplementary material for: Eugenol as a potential adjuvant therapy for gingival squamous cell carcinoma
Source: Sci Rep. 2024 May 13;14:10958. doi: 10.1038/s41598-024-60754-8 (PMC11091204; doi:10.1038/s41598-024-60754-8)
Supplement: Supplementary file 7 — Supplementary Table 2. [file 41598_2024_60754_MOESM7_ESM.pptx]

## Slide 1
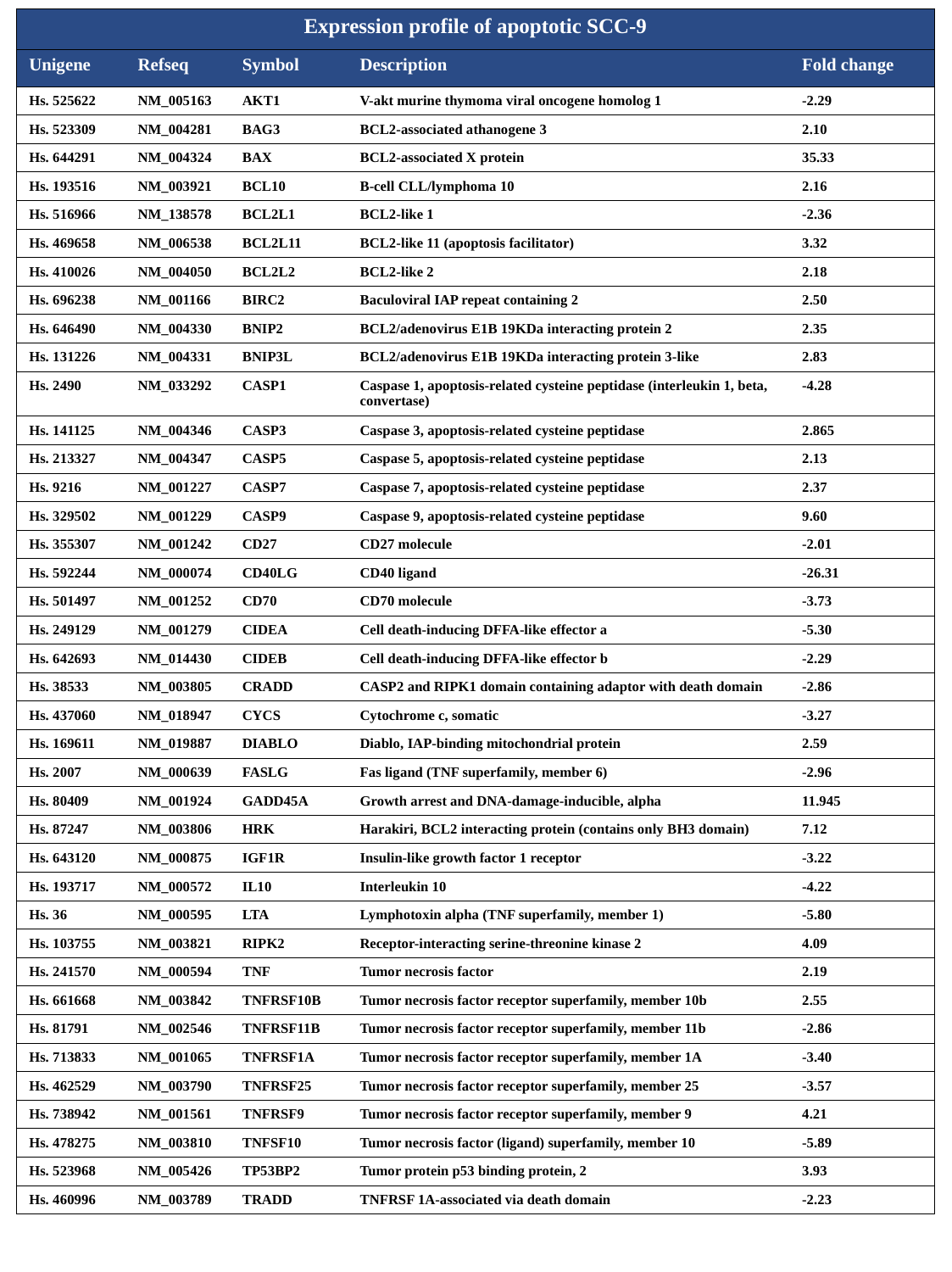

| Expression profile of apoptotic SCC-9 | | | | |
| --- | --- | --- | --- | --- |
| Unigene | Refseq | Symbol | Description | Fold change |
| Hs. 525622 | NM\_005163 | AKT1 | V-akt murine thymoma viral oncogene homolog 1 | -2.29 |
| Hs. 523309 | NM\_004281 | BAG3 | BCL2-associated athanogene 3 | 2.10 |
| Hs. 644291 | NM\_004324 | BAX | BCL2-associated X protein | 35.33 |
| Hs. 193516 | NM\_003921 | BCL10 | B-cell CLL/lymphoma 10 | 2.16 |
| Hs. 516966 | NM\_138578 | BCL2L1 | BCL2-like 1 | -2.36 |
| Hs. 469658 | NM\_006538 | BCL2L11 | BCL2-like 11 (apoptosis facilitator) | 3.32 |
| Hs. 410026 | NM\_004050 | BCL2L2 | BCL2-like 2 | 2.18 |
| Hs. 696238 | NM\_001166 | BIRC2 | Baculoviral IAP repeat containing 2 | 2.50 |
| Hs. 646490 | NM\_004330 | BNIP2 | BCL2/adenovirus E1B 19KDa interacting protein 2 | 2.35 |
| Hs. 131226 | NM\_004331 | BNIP3L | BCL2/adenovirus E1B 19KDa interacting protein 3-like | 2.83 |
| Hs. 2490 | NM\_033292 | CASP1 | Caspase 1, apoptosis-related cysteine peptidase (interleukin 1, beta, convertase) | -4.28 |
| Hs. 141125 | NM\_004346 | CASP3 | Caspase 3, apoptosis-related cysteine peptidase | 2.865 |
| Hs. 213327 | NM\_004347 | CASP5 | Caspase 5, apoptosis-related cysteine peptidase | 2.13 |
| Hs. 9216 | NM\_001227 | CASP7 | Caspase 7, apoptosis-related cysteine peptidase | 2.37 |
| Hs. 329502 | NM\_001229 | CASP9 | Caspase 9, apoptosis-related cysteine peptidase | 9.60 |
| Hs. 355307 | NM\_001242 | CD27 | CD27 molecule | -2.01 |
| Hs. 592244 | NM\_000074 | CD40LG | CD40 ligand | -26.31 |
| Hs. 501497 | NM\_001252 | CD70 | CD70 molecule | -3.73 |
| Hs. 249129 | NM\_001279 | CIDEA | Cell death-inducing DFFA-like effector a | -5.30 |
| Hs. 642693 | NM\_014430 | CIDEB | Cell death-inducing DFFA-like effector b | -2.29 |
| Hs. 38533 | NM\_003805 | CRADD | CASP2 and RIPK1 domain containing adaptor with death domain | -2.86 |
| Hs. 437060 | NM\_018947 | CYCS | Cytochrome c, somatic | -3.27 |
| Hs. 169611 | NM\_019887 | DIABLO | Diablo, IAP-binding mitochondrial protein | 2.59 |
| Hs. 2007 | NM\_000639 | FASLG | Fas ligand (TNF superfamily, member 6) | -2.96 |
| Hs. 80409 | NM\_001924 | GADD45A | Growth arrest and DNA-damage-inducible, alpha | 11.945 |
| Hs. 87247 | NM\_003806 | HRK | Harakiri, BCL2 interacting protein (contains only BH3 domain) | 7.12 |
| Hs. 643120 | NM\_000875 | IGF1R | Insulin-like growth factor 1 receptor | -3.22 |
| Hs. 193717 | NM\_000572 | IL10 | Interleukin 10 | -4.22 |
| Hs. 36 | NM\_000595 | LTA | Lymphotoxin alpha (TNF superfamily, member 1) | -5.80 |
| Hs. 103755 | NM\_003821 | RIPK2 | Receptor-interacting serine-threonine kinase 2 | 4.09 |
| Hs. 241570 | NM\_000594 | TNF | Tumor necrosis factor | 2.19 |
| Hs. 661668 | NM\_003842 | TNFRSF10B | Tumor necrosis factor receptor superfamily, member 10b | 2.55 |
| Hs. 81791 | NM\_002546 | TNFRSF11B | Tumor necrosis factor receptor superfamily, member 11b | -2.86 |
| Hs. 713833 | NM\_001065 | TNFRSF1A | Tumor necrosis factor receptor superfamily, member 1A | -3.40 |
| Hs. 462529 | NM\_003790 | TNFRSF25 | Tumor necrosis factor receptor superfamily, member 25 | -3.57 |
| Hs. 738942 | NM\_001561 | TNFRSF9 | Tumor necrosis factor receptor superfamily, member 9 | 4.21 |
| Hs. 478275 | NM\_003810 | TNFSF10 | Tumor necrosis factor (ligand) superfamily, member 10 | -5.89 |
| Hs. 523968 | NM\_005426 | TP53BP2 | Tumor protein p53 binding protein, 2 | 3.93 |
| Hs. 460996 | NM\_003789 | TRADD | TNFRSF 1A-associated via death domain | -2.23 |
